# Whole genome sequencing of clinical samples reveals extensively drug resistant tuberculosis (XDR TB) strains from the Beijing lineage in Nigeria, West Africa

**DOI:** 10.1038/s41598-021-96956-7

**Published:** 2021-08-30

**Authors:** Idowu B. Olawoye, Jessica N. Uwanibe, Chioma N. Kunle-Ope, Olabisi F. Davies-Bolorunduro, Temitope A. Abiodun, Rosemary A. Audu, Babatunde L. Salako, Christian T. Happi

**Affiliations:** 1 African Centre of Excellence for Genomics of Infectious Diseases (ACEGID), Redeemer’s University, Ede, Osun State Nigeria; 2 Department of Biological Sciences, Faculty of Natural Sciences, Redeemer’s University, Ede, Osun State Nigeria; 3 Centre for Tuberculosis Research (CTBR), Microbiology Department, Nigerian Institute of Medical Research (NIMR), Yaba, Lagos State Nigeria

**Keywords:** Computational biology and bioinformatics, Genomics, Public health, Genomic analysis

## Abstract

Multi-drug (MDR) and extensively drug-resistant (XDR) tuberculosis (TB) continues to be a global public health problem especially in high TB burden countries like Nigeria. Many of these cases are undetected and go on to infect high risk individuals. Clinical samples from positive rifampicin resistant Xpert®MTB/Rif assay were subjected to direct whole genome sequencing and bioinformatics analysis to identify the full antibiotics resistance and lineage profile. We report two (2) XDR TB samples also belonging to the East-Asian/Beijing family of lineage 2 *Mycobacterium tuberculosis* complex from clinical samples in Nigeria. Our findings further reveal the presence of mutations that confer resistance to first-line drugs (rifampicin, isoniazid, ethambutol and pyrazanimide), second-line injectables (capreomycin, streptomycin, kanamycin and/or amikacin) and at least one of the fluoroquinolones (ofloxacin, moxifloxacin, levofloxacin and/or ciprofloxacin) in both samples. The genomic sequence data from this study not only provide the first evidence of XDR TB in Nigeria and West Africa, but also emphasize the importance of WGS in accurately detecting MDR and XDR TB, to ensure adequate and proper management treatment regimens for affected individuals. This will greatly aid in preventing the spread of drug resistance TB in high burden countries.

## Introduction

*Mycobacterium tuberculosis* complex (MTBC) which is responsible for tuberculosis (TB) remains a global public health concern especially in low-income countries as it is the leading cause of death from a bacterial infectious disease^1^. In 2019, it was estimated that ten million people worldwide contracted the tubercle disease and about 1.2 million (HIV negative) of these people died as a result of complications that arose from TB^1^. MTBC is grouped into different lineages, known as *M. tuberculosis *sensu stricto (lineage 1, 2, 3, 4 and, 7), *M. africanum* (lineage 5 and 6), the recently discovered lineages 8, 9^2,3^, and, several other animal-associated habitat (*M. bovis, M.microti, M.pinnipedii, M. orygis* and, others)^4^. Some of these lineages are geographically restricted, such as lineages 5, 6, 7 and, 9^2,5,6^, whilst some are globally distributed such as lineages 1, 2, 3 and, 4^7^.

Lineage 2, also known as the Beijing lineage is associated with the increased spread of multi-drug resistant TB in Eurasia, and has since spread across the world in successive waves due to human migration^8^. Genome analysis have reported multiple introductions of the Beijing lineage in Africa dating back to 300 years^9^. In addition, the Beijing lineage have been reported in the West African region, using spoligotyping and whole genome sequencing^10–12^, but with no report in Nigeria yet using molecular techniques^13,14^.

Multi-drug resistant TB (MDR TB) is the type of TB bacteria that is resistant to two of the most important drugs to treat TB also known as first-line drugs: isoniazid (INH) and rifampicin (RIF). The updated definition of extensively drug-resistant TB (XDR TB) is a rare type of MDR TB that is resistant to INH, RIF and any fluoroquinolone, and at least one additional Group A drug (levofloxacin, moxifloxacin bedaquiline and/or, linezolid) which are the most powerful second-line drugs used for drug resistant TB management^15^. The emergence of drug resistant and extensively drug resistant MTBC worldwide has largely affected public health and global economy, as many cases go unreported or undetected in many low resource settings and cause further spread and misdiagnosis^16^. In 2019, the incidence of MDR TB resistant to rifampicin (RR-TB) in Nigeria was estimated at 21,000 with 16 laboratory confirmed XDR TB cases, with reports that about 2% of global MDR TB cases acquire multiple resistance to antibiotics to become XDR TB^1^. This statistics may be underplayed as many high burden TB countries do not have diagnostic capacities to capture the real scenario, as such it is crucial to use cutting edge technologies to screen drug-resistant (DR) or MDR TB samples for the presence of XDR acquiring mutations in these countries^17^.

Just as many other West African countries, Nigeria has deployed rapid molecular techniques to detect RR-TB and MDR TB by using Xpert®MTB/Rif assay (Cephid, USA) and GenoType MTDRplus Line Probe Assay (Hain Lifescience GmbH, Germany)^18^. However, these techniques come with limitations when compared to whole genome sequencing (WGS), as the latter can detect drug resistant mutations outside the target zone of rapid tests and produce much more reliable and robust drug resistance profile of TB^19^.

In this study, we use whole genome sequencing to investigate TB positive clinical samples that were confirmed RR-TB with Xpert®MTB/Rif assay (Cephid, USA). We also use bioinformatics to classify the bacteria into lineages in order to identify the strains or clonal complexes present in Nigeria.

## Results

### Patient demography and molecular drug resistance outcome

Out of ten (10) de-identified patients, 20% (2/10) were females and 20% (2/10) tested positive to HIV. The ages of the patients were between 20 and 49 years. All samples (100%) tested positive to resistance to RIF using Xpert®MTB/Rif assay and 20% tested positive to resistance to INH and none of the samples tested positive to fluoroquinolones (FLQ) or second-line drug injectables (kanamycin, amikacin, capreomycin and/or, vancomycin) using Hain Lifescience GenoType MTBDR*plus* and MTBDR*sl* line probe assays (Table 1).

**Table 1:** Patient demography and rapid diagnostic molecular drug resistance outcome.

	Sex	Age	HIV Status	INH	RIF	FLQ	KAN/AMK/CAP/VAN	Low level KAN	Treatment type
1	Male	48	Negative	S	R	S	S	S	New TB case
2	Male	26	Negative	S	R	S	S	S	New TB case
3	Male	34	Negative	S	R	S	S	S	New TB case
4	Male	45	Negative	S	R	S	S	S	New TB case
5	Male	49	Negative	S	R	S	S	S	New TB case
6	Female	NA	Positive	S	R	S	S	S	New TB case
7	Male	25	Negative	S	R	S	S	S	New TB case
8	Female	31	Positive	R	R	S	S	S	New TB case
9	Male	23	Negative	S	R	S	S	S	New TB case
10	Male	20	Negative	R	R	S	S	S	New TB case

*NA* not available, *S* sensitive, *R* resistant, *FLQ* fluoroquinolones, *KAN* kanamycin, *AMK* amikacin, *CAP* capreomycin, *VAN* vancomycin.

### Whole genome sequencing and quality control

Out of 10 samples that were quantified using Qubit and Agilent 2100 Bioanalyzer, only 4 libraries were prioritized for whole genome sequencing as they had high concentration of DNA that passed the loading concentration on the Illumina iSeq 100. Two (2) out of four (4) samples passed the 20× average depth, and 220 genomes downloaded from SRA passed the quality control (QC) threshold used for all samples. The average depth of the two newly sequenced TB samples from Nigeria (TB3qc and TB8qc) were 71.66× and 63.59× using SAMtools. The average depth of the other SRA genomes in this study were within ± 40× of the two newly sequenced samples.

### Phylogenetic analysis and in-silico drug resistance profiling of TB samples

**Figure 1:**
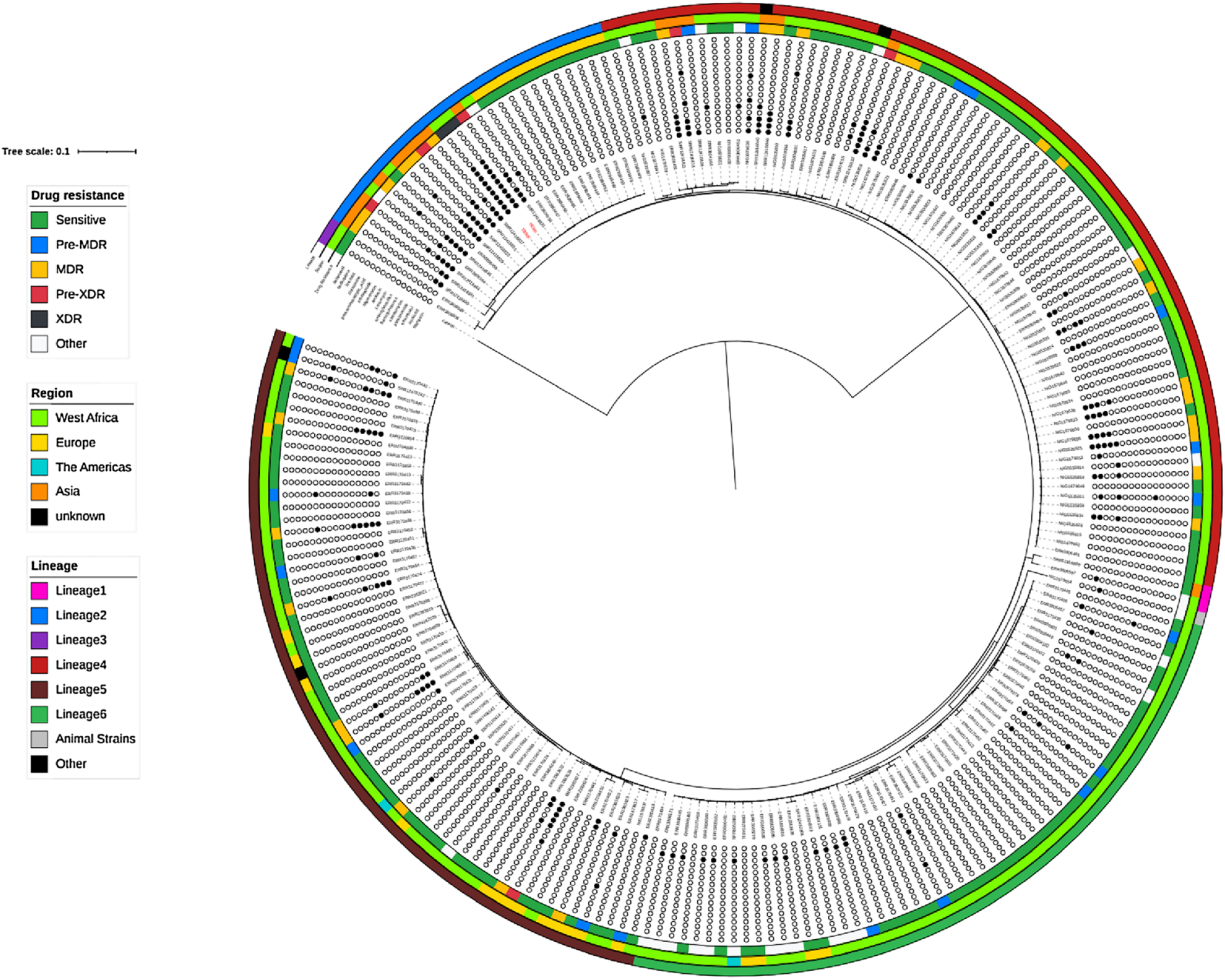
Maximum likelihood phylogeny tree of MTBC samples inferred from their core genomes with lineage classification, region where samples were collected and drug resistance profile. The samples coloured red are genomes generated from this study.

Geographical location of all MTBC genomes were retrieved from SRA and grouped into: The Americas, Europe, Asia and, West Africa. Phylogenetic analysis of core genomes containing SNPs of all samples in this study that passed QC steps showed that TB3qc and TB8qc clustered alongside other global lineage 2 genomes as seen in Fig. 1.

Drug resistance profiling of TB3qc and TB8qc classified them as XDR TB and lineage classification as Lineage 2.2.1 also known as Beijing or East-Asian lineage. Analysis of drug mutations associated with resistance to anti-TB drugs show that these samples have mutations against all first and second line drug classes (Table 2).

**Table 2:** In-silico drug-resistant analysis of MTBC samples and Lineage report.

		Sample ID	
		TB3qc	TB8qc
**Lineage, Family and Spoligotype**		Lineage 2.2.1; East-Asian (Beijing)	Lineage 2.2.1; East-Asian (Beijing)
**Region of difference (RD)**		RD105, RD207 and RD181	RD105, RD207 and RD181
**Drug resistance group**		XDR TB	XDR TB
**Drug genotypic resistance (TB-Profiler)**			
Rifampicin	*rpoB*	S450L	S450L
Isoniazid	*katG*	S315T, c1561 del	S315T
Pyrazinamide	*pncA*	D12G	D12G
Ethambutol	*embB*	Q497R	Q497R
Streptomycin	*rpsL*	K43R	K43R
	*rrs*	c799t	c799t, g888a
Fluoroquinolones	*gyrA*	A90V, S91P	A90V, S91P
Amikacin	*rrs*	c1402a	c1402a, r1484t
Kanamycin	*rrs*	c1402a	c1402a, r1484t
Capreomycin	*rrs*	c1402a	c1402a, r1484t
Cycloserine	*ald*	–	–
Bedaquiline	*Rv0678*	–	–
Delamanid	*ddn*	–	–
Clofazimine	*Rv0678*	–	–
**Drug genotypic resistance (Mykrobe)**			
Amikacin	*rrs*	–	–
Capreomycin	*rrs*	C1402X	–
Ciprofloxacin	*gyrA*	A90V, S91P	A90V, S91P
Ethambutol	*embB*	Q497R	Q497R
Isoniazid	*katG*	S315G	S315G
Kanamycin	*rrs*	–	–
Moxifloxacin	*gyrA*	A90V, S91P	A90V, S91P
Ofloxacin	*gyrA*	A90V, S91P	A90V, S91P
Pyrazinamide	*pncA*	D12G	D12G
Rifampicin	*rpoB*	S450L	S450L
Streptomycin	*rpsL*	K43R	K43R
**Drug genotypic resistance (MTB Resistance Sniffer)**			
Amikacin		0.25 + / − 0.06 [26 sites]	0.25 + / − 0.06 [26 sites]
Capreomycin		0.16 + / − 0.04 [27 sites]	0.16 + / − 0.04 [27 sites]
Cycloserin		0.06 + / − 0.02 [22 sites]	0.06 + / − 0.02 [22 sites]
Ethambutol		0.17 + / − 0.05 [26 sites]	0.17 + / − 0.05 [26 sites]
Ethionamide		0.29 + / − 0.08 [23 sites]	0.28 + / − 0.08 [21 sites]
Floroquinolones		0.41 + / − 0.07 [43 sites]	0.42 + / − 0.07 [40 sites]
Isoniazid		0.81 + / − 0.1 [26 sites]	0.81 + / − 0.1 [26 sites]
Kanamycin		0.79 + / − 0.12 [25 sites]	0.73 + / − 0.12 [22 sites]
Ofloxacin		0.27 + / − 0.07 [26 sites]	0.27 + / − 0.07 [26 sites]
Para-amino salicylic acid		0.48 + / − 0.1 [28 sites]	0.5 + / − 0.11 [27 sites]
Pyrazinamide		0.27 + / − 0.07 [26 sites]	0.28 + / − 0.07 [25 sites]
Rifampicin		0.41 + / − 0.08 [26 sites]	0.41 + / − 0.08 [26 sites]
Streptomycin		0.92 + / − 0.09 [26 sites]	0.92 + / − 0.09 [26 sites]

Full drug resistance profile of TB3qc and TB8qc from TB-profiler and Mykrobe predictor in CSV and JSON files containing genome position, locus tag, estimated fraction, coverage of genotype and, other mutations associated with drug resistance are available in Supplementary Data 1, 2, 3 and 4 such as P239L and L707I substitution mutations in *katG*.

TB3qc and TB8qc drug susceptibility phenotypic test outcome classified them as drug resistant and pre-XDR respectively and the patients treatment outcome was followed up from the Lagos State MDR TB treatment centre (Table 3).

**Table 3:** Laboratory results, phenotypic drug sensitivity test and treatment outcome from the two sequenced samples.

	Smear result	Culture result		First line drug			Second line drug					Treatment outcome
		Solid	Liquid	INH	RIF	EMB	LEV	MOX	KAN	AMK	CAP	
TB3qc	3+	POS	POS	S	R	S	S	S	S	S	S	Stopped treatment
TB8qc	2+	POS	POS	R	R	R	R	R	S	S	S	DEAD

*POS* positive, *INH* isoniazid, *RIF* rifampicin, *EMB* ethambutol, *LEV* levofloxacin, *MOX* moxifloxacin, *KAN* kanamycin, *AMK* amikacin, *CAP* capreomycin.

## Discussion

In this study, we were able to use next generation sequencing (NGS) technology to identify the presence of the Beijing family of MTBC lineage 2 and extensively drug-resistant tuberculosis in Nigeria for the first time. Maximum likelihood phylogeny from variable regions in core genome alignments and also using characteristic regions of differences such as the presence of RD105, RD207 and, RD181 in the genome grouped them as lineage 2.2.1. In addition, these genomes were classified as extensively drug-resistant TB (XDR TB) as they have defining mutations that confer resistance to all first-line drugs (isoniazid, rifampicin, ethambutol and, pyrazinamide), at least one second-line anti-TB injectables or aminoglycosides (capreomycin, streptomycin, kanamycin and/or, amikacin) and at least one fluoroquinolone (ofloxacin, moxifloxacin, levofloxacin and/or, ciprofloxacin) (Table 2). Resistance R-value below 0.3 for drug classes analyzed with Resistance Sniffer implies that the strain has higher likelihood to be sensitive to said drug, with number of resistance sites present in the genome in square brackets (Table 2).

The presence of the Beijing lineage in Nigeria is no surprise as it has been detected in Benin Republic, which shares the western border with Nigeria^10^ and more so, it may have resulted from the continuous increase of economic relations between Nigeria and China over the past decades^20^. The East-Asian or Beijing lineage of MTBC is well known for multi-drug resistance, increased virulence and, fast disease progression unlike other MTBC lineages^8,9^, with another study seeing a level of association with HIV^21^.

In our findings, one of the patients who tested positive to HIV was diagnosed as MDR TB whilst the other was diagnosed as drug-resistant TB as both samples tested negative to second-line drugs using rapid molecular tests which many TB labs in Nigeria solely rely on. However, both samples were confirmed to be XDR TB with whole genome sequence analysis. The reason for this disparity is that some false negative results can be produced by the MTBDR*sl* strip-based assay as some second-line drug mutations may not be built in the panel which can be detected using whole genome sequence data by screening mutations that are not part of the LPA as seen in Feliciano et al.^22^. After WGS, phenotypic drug susceptibility test (DST) was done to correlate the results from rapid molecular tests and TB3qc still remained drug resistant TB, while TB8qc tested as pre-XDR TB.

This infers that a number of XDR TB cases will go undetected using LPAs as we can see in the Mykrobe predictor JSON output file for TB3qc (Supplementary Data 2) where the allele responsible for resistance to isoniazid has 100% coverage and a median depth of 39 and R-value of 0.81 from Resistance Sniffer (Table 2). However, this sample tested sensitive to isoniazid through LPA and DST test. Other advantages of WGS is that new drug-resistant mutations can also be detected across the entire genome and not just target genes as seen under ‘other variants report’ section in TB-Profiler CSV file outputs of TB3qc and TB8qc (Supplementary Data 1 and 3) where other drug resistance mutations present in the genomes are listed. Furthermore, antimicrobial resistance (AMR) tools used for genome analysis are constantly being improved, such as one of the software used in this research which was developed with a drug resistance database extracted from over 17,000 MTBC strains with their phenotypic and genotypic data^23^.

Following up on the patients treatment outcome from the MDR TB treatment centre in Lagos State revealed that the patient who tested positive to HIV and had an XDR TB died as a result of complications from both infections, while the other patient stopped treatment due to severe side effects arising from the treatment regimen. This patient can potentially go on to infect others with this highly infectious and drug resistant strain of TB.

The major discovery in this study is that we were able to identify the presence of the Beijing family of lineage 2 MTBC in Nigeria for the first time using WGS from direct clinical samples. This shows that there is more genetic diversity of MTBC in Nigeria than presently known. In addition to that, we were also able to characterize two (2) XDR TB from drug-resistant and multi-drug resistant TB strains that were initially tested using LPA and Xpert®MTB/Rif assays. This shows the high resolution of WGS for AMR detection in clinical diagnosis and public health. In addition, WGS analysis show that the XDR TB samples detected in this study are sensitive to new and repurposed drugs such as delamanid, bedaquiline and cycloserine, which implies that they can be managed with either of these.

With the falling cost of NGS technologies nowadays, WGS has the potential to bolster clinical diagnosis of TB in low-to-middle income countries and high TB burden regions as it will greatly improve the accuracy of antimicrobial resistance (AMR) detection.

Although this study is limited by the small sample size, it provide new insights into TB genetic diversity in Nigeria and West Africa, despite budgetary restrictions to perform WGS.

## Conclusion

In this study, we show the robustness of WGS in public health and diagnosis of drug resistance tuberculosis. The data generated from this work shows how extensively drug resistance could be missed when using routine rapid molecular diagnosis assays only. Bioinformatics analysis can reveal sensitivity or resistance to new drugs by checking for the presence or absence of mutations responsible for such phenotypic reactions. We conclude that the integration of NGS platforms into the established, but siloed, clinical diagnosis, to support the detection of extensively drug resistant tuberculosis in high burden countries, will not only help in suggesting treatment options to MDR/XDR TB patients, but will also provide exciting opportunities for public health surveillance.

## Methods

### Sample collection, DNA extraction and Molecular drug resistance assay

Random sputum samples (n = 10) that were collected in the month of July, 2019 from patients who were referred for diagnosis at the Centre of Tuberculosis Research (CTBR), a TB reference laboratory in Lagos State. Patients who had severe TB symptoms and tested positive with Xpert®MTB/RIF (Cephid, USA) to detect the presence of MTBC and resistance to RIF were recruited for this study. These samples were further subjected to DNA extraction using the GenoLyse kit (Hain Lifescience GmbH, Germany). The genomic bacterial DNA further underwent Line Probe Assay (LPA) using GenoType MTBDR*plus* version 2.0 (Hain Lifescience GmbH, Germany) to detect samples resistant to RIF and INH. Second-line drug resistance molecular test was done using GenoType MTBDR*sl* VER 2.0 (Hain Lifescience GmbH, Germany) strip-based assay. Samples of interest were cultured on Lowenstein Jensen (LJ) media and Middlebrook 7H9 broth using BACTEC™ MGIT machine (BD, Erembodegem, Belgium) and tested using proportion method for phenotypic drug susceptibility with first and second line drugs.

These molecular processes were carried out at CTBR, Microbiology Department, Nigerian Institute of Medical Research (NIMR), Lagos.

### Library preparation and whole genome sequencing

DNA extracts were quantified using Qubit fluorometer (ThermoFisher Scientific) using the dsDNA High sensitivity assay and diluted to 0.2 ng/ul. Sequencing libraries were prepared using Nextera XT DNA preparation kit (Illumina, USA) and libraries were quantified with Qubit fluorometer and the library length was estimated using the Agilent 2100 Bioanalyzer (Agilent Technologies, USA). Library preparation protocol was adopted from the US CDC PulseNet SOP^24^. Individual libraries were loaded on the Illumina iSeq 100 (Illumina, USA) with 151 cycles to generate paired end reads using the manufacturer’s instructions (Illumina, USA). TB genome sequencing was done at the African Centre of Excellence for Genomics of Infectious Diseases (ACEGID), Redeemer’s University, Nigeria.

### Bioinformatics analysis

The paired-end reads were demultiplexed using the GenerateFASTQ module version 2.0 on the iSeq 100 Software System Suite local run manager. 230 MTBC genomes were retrieved from NCBI SRA using sra-toolkit v2.10.9 (https://github.com/ncbi/sra-tools) with paired-end reads and Illumina platform as the selection criteria from the following BioProjects: PRJEB15857, PRJEB25506, PRJNA300846, PRJEB28842, PRJNA633244, PRJNA480117, PRJEB27244, PRJEB36076, PRJNA534674 and, PRJNA655747.

All reads were processed with the Bacterial Genome Pipeline (BAGEP)^25^, which does quality control on raw reads, taxonomic classification and variants detection by mapping reads to the reconstructed ancestral MTBC sequence^26^ with minimum base quality set at 20, minimum site depth for calling alleles set at 10 (default value) and also excluding duplicate reads. Average coverage of the genomes were calculated using SAMtools v1.12^27^ and genomes that are ≤ 20× was excluded from further downstream analysis.

### Drug resistance prediction and lineage classification

TBProfiler v3.0.4^23^ was used to analyze all 222 genomes in-silico to infer phylogenetic lineages and drug resistance profiles using small variants and big deletions associated with drug resistance that are present in a robust library of recently updated mutation database which contains new anti-TB drugs such as cycloserine, delamanid, bedaquiline, clofazimine, para-aminosalicylic acid, linezolid and ethionamide [commit b2af444 on 21st December 2020 (https://github.com/jodyphelan/tbdb)] using freebayes^28^ as the variant calling option. In order to validate the results, Mykrobe predictor^29^ and Resistance Sniffer^30^ was used to analyze the newly sequenced Nigerian samples for comparison.

### Phylogenetic reconstruction

Outputs from all the variant call analysis with Snippy were combined to produce core genome alignment of all 223 samples (including *Mycobacterium canettii* NC_015848.1) were generated and aligned using Snippy-core (https://github.com/tseemann/snippy) by excluding regions such as PPE, PE-PGRS, insertion sequences, phages, repeat sequences and, regions that are at least 50 bp long. The core genome alignment containing polymorphisms was used to infer a maximum-likelihood phylogenetic tree using IQTREE^31^ v1.6.12 general time reversible model (GTR) and ultrafast bootstrap value of 10,000. The phylogenetic tree was rooted to *M. canettii* and annotated with Interactive tree of life (iTOL) v5^32^.

### Ethical approval

The study was performed in accordance with the Declaration of Helsinki, and the protocol was approved by the ethical review boards of Redeemer’s University (Osun State, Nigeria) (RUN-IREC/19/012) and Nigerian Institute of Medical Research (Lagos State, Nigeria) (IRB/19/053). Sputum samples used for this study were obtained under a waiver of consent granted by the Institutional Review Board of the Nigerian Institute of Medical Research (ref: IRB/19/053).

## Supplementary Information

Supplementary Information 1.

Supplementary Information 2.

Supplementary Information 3.

Supplementary Information 4.

Supplementary Information 5.

## Data Availability

Raw sequence data generated in this study are available from the National Centre for Biotechnology Information (NCBI) Sequence Read Archive (SRA) under the Project Accession No. PRJNA725394 (https://www.ncbi.nlm.nih.gov/bioproject/PRJNA725394/).
