# Supplementary material for: Whole genome sequencing of clinical samples reveals extensively drug resistant tuberculosis (XDR TB) strains from the Beijing lineage in Nigeria, West Africa
Source: Sci Rep. 2021 Aug 30;11:17387. doi: 10.1038/s41598-021-96956-7 (PMC8405707; doi:10.1038/s41598-021-96956-7)
Supplement: Supplementary file 1 — Supplementary Information 1. [file 41598_2021_96956_MOESM1_ESM.docx]

# Supporting information

**Supplementary Data 1. TB3qc TBprofiler complete results**

**Supplementary Data 2. TB3qc Mykrobe complete results**

**Supplementary Data 3. TB8qc TBprofiler complete results**

**Supplementary Data 4. TB8qc Mykrobe complete results**
