# Supplementary material for: Whole genome sequencing of clinical samples reveals extensively drug resistant tuberculosis (XDR TB) strains from the Beijing lineage in Nigeria, West Africa
Source: Sci Rep. 2021 Aug 30;11:17387. doi: 10.1038/s41598-021-96956-7 (PMC8405707; doi:10.1038/s41598-021-96956-7)
Supplement: Supplementary file 2 — Supplementary Information 2. [file 41598_2021_96956_MOESM2_ESM.pdf]

{
species": {
"susceptibility": {
"Ofloxacin": {
"predict": "R

called_by": {
"gyrA_A90V-GCG7569GTG": {
"variant": null,
"genotype": [
1,
1
],
"genotype_likelihoods": [
-4290.611436662613,
-99999999,
-46.94967162259618
],
"info": {
"coverage": {
"reference": {
"percent_coverage": 0.0,
"median_depth": 0,
"min_non_zero_depth": 0,
"kmer_count": 0,
"klen": 21
},
"alternate": {
"percent_coverage": 100.0,
"median_depth": 34,
"min_non_zero_depth": 30,
"kmer_count": 645,
"klen": 20
}
},
"expected_depths": [
39
],
"contamination_depths": [],
"filter": [],
"conf": 4244
},
"_cls": "Call.VariantCall

}

gyrA_S91P-TCG7572CCG": {
"variant": null,
"genotype": [
1,
1
],
"genotype_likelihoods": [
-4337.755190291881,
-99999999,
-43.144937925898645
],
"info": {
"coverage": {
"reference": {
"percent_coverage": 0.0,
"median_depth": 0,
"min_non_zero_depth": 0,
"kmer_count": 0,
"klen": 21
},
"alternate": {
"percent_coverage": 100.0,
"median_depth": 33,
"min_non_zero_depth": 28,
"kmer_count": 665,
"klen": 21
}
},
"expected_depths": [
39
],
"contamination_depths": [],
"filter": [],
"conf": 4295
},
"_cls": "Call.VariantCall
}
}
}
Moxifloxacin": {
"predict": "R

```
called_by": {
    "gyrA_A90V-GCG7569GTG": {
        "variant": null,
        "genotype": [
            1,
            1
        ],
        "genotype_likelihoods": [
            -4290.611436662613,
            -99999999,
            -46.94967162259618
        ],
        "info": {
            "coverage": {
                "reference": {
                    "percent_coverage": 0.0,
                    "median_depth": 0,
                    "min_non_zero_depth": 0,
                    "kmer_count": 0,
                    "klen": 21
                },
                "alternate": {
                    "percent_coverage": 100.0,
                    "median_depth": 34,
                    "min_non_zero_depth": 30,
                    "kmer_count": 645,
                    "klen": 20
                }
            },
            "expected_depths": [
                39
            ],
            "contamination_depths": [],
            "filter": [],
            "conf": 4244
        },
        "_cls": "Call.VariantCall
    }
```

gyrA_S91P-TCG7572CCG": {
"variant": null,
"genotype": [
1,
1
],
"genotype_likelihoods": [
-4337.755190291881,
-99999999,
-43.144937925898645
],
"info": {
"coverage": {
"reference": {
"percent_coverage": 0.0,
"median_depth": 0,
"min_non_zero_depth": 0,
"kmer_count": 0,
"klen": 21
},
"alternate": {
"percent_coverage": 100.0,
"median_depth": 33,
"min_non_zero_depth": 28,
"kmer_count": 665,
"klen": 21
}
},
"expected_depths": [
39
],
"contamination_depths": [],
"filter": [],
"conf": 4295
},
"_cls": "Call.VariantCall
}
}
}
Isoniazid": {
"predict": "R

called_by": {
"katG_S315G-GCT2155167GGT": {
"variant": null,
"genotype": [
1,
1
],
"genotype_likelihoods": [
-4672.233848007324,
-99999999,
-30.903794845738865
],
"info": {
"coverage": {
"reference": {
"percent_coverage": 0.0,
"median_depth": 0,
"min_non_zero_depth": 0,
"kmer_count": 0,
"klen": 21
},
"alternate": {
"percent_coverage": 100.0,
"median_depth": 39,
"min_non_zero_depth": 32,
"kmer_count": 741,
"klen": 20
}
},
"expected_depths": [
39
],
"contamination_depths": [],
"filter": [],
"conf": 4641
},
"_cls": "Call.VariantCall
}
}
}
Kanamycin": {
"predict": "S

}
Ethambutol": {
"predict": "R

called_by": {
"embB_Q497R-CAG4248002CGG": {
"variant": null,
"genotype": [
1,
1
],
"genotype_likelihoods": [
-5204.863923415898,
-99999999,
-25.15674404283727
],
"info": {
"coverage": {
"reference": {
"percent_coverage": 10.0,
"median_depth": 0,
"min_non_zero_depth": 1,
"kmer_count": 2,
"klen": 21
},
"alternate": {
"percent_coverage": 100.0,
"median_depth": 48,
"min_non_zero_depth": 42,
"kmer_count": 910,
"klen": 20
}
},
"expected_depths": [
39
],
"contamination_depths": [],
"filter": [],
"conf": 5180
},
"_cls": "Call.VariantCall
}
}

}
Streptomycin": {
"predict": "R

called_by": {
"rpsL_K43R-AAG781686AGG": {
"variant": null,
"genotype": [
1,
1
],
"genotype_likelihoods": [
-2914.920854490103,
-99999999,
-129.77218775526921
],
"info": {
"coverage": {
"reference": {
"percent_coverage": 30.0,
"median_depth": 0,
"min_non_zero_depth": 5,
"kmer_count": 36,
"klen": 21
},
"alternate": {
"percent_coverage": 100.0,
"median_depth": 23,
"min_non_zero_depth": 11,
"kmer_count": 440,
"klen": 20
}
},
"expected_depths": [
39
],
"contamination_depths": [],
"filter": [],
"conf": 2785
},
"_cls": "Call.VariantCall
}
}

}
Ciprofloxacin": {
"predict": "R

called_by": {
"gyrA_A90V-GCG7569GTG": {
"variant": null,
"genotype": [
1,
1
],
"genotype_likelihoods": [
-4290.611436662613,
-99999999,
-46.94967162259618
],
"info": {
"coverage": {
"reference": {
"percent_coverage": 0.0,
"median_depth": 0,
"min_non_zero_depth": 0,
"kmer_count": 0,
"klen": 21
},
"alternate": {
"percent_coverage": 100.0,
"median_depth": 34,
"min_non_zero_depth": 30,
"kmer_count": 645,
"klen": 20
}
},
"expected_depths": [
39
],
"contamination_depths": [],
"filter": [],
"conf": 4244
},
"_cls": "Call.VariantCall
}

gyrA_S91P-TCG7572CCG": {
"variant": null,
"genotype": [
1,
1
],
"genotype_likelihoods": [
-4337.755190291881,
-99999999,
-43.144937925898645
],
"info": {
"coverage": {
"reference": {
"percent_coverage": 0.0,
"median_depth": 0,
"min_non_zero_depth": 0,
"kmer_count": 0,
"klen": 21
},
"alternate": {
"percent_coverage": 100.0,
"median_depth": 33,
"min_non_zero_depth": 28,
"kmer_count": 665,
"klen": 21
}
},
"expected_depths": [
39
],
"contamination_depths": [],
"filter": [],
"conf": 4295
},
"_cls": "Call.VariantCall
}
}
}
Pyrazinamide": {
"predict": "r

```
called_by": {
    "pncA_D12G-GTC2289206GCC": {
        "variant": null,
        "genotype": [
            0,
            1
        ],
        "genotype_likelihoods": [
            -1903.656725557816,
            -158.99583713302795,
            -276.22421683691135
        ],
        "info": {
            "coverage": {
                "reference": {
                    "percent_coverage": 100.0,
                    "median_depth": 8,
                    "min_non_zero_depth": 4,
                    "kmer_count": 132,
                    "klen": 21
                },
                "alternate": {
                    "percent_coverage": 100.0,
                    "median_depth": 27,
                    "min_non_zero_depth": 24,
                    "kmer_count": 525,
                    "klen": 20
                }
            },
            "expected_depths": [
                39
            ],
            "contamination_depths": [],
            "filter": [],
            "conf": 1745
        },
        "_cls": "Call.VariantCall
    }
  }
}
Rifampicin": {
  "predict": "R
```

called_by": {
"rpoB_S450L-TCG761154TTG": {
"variant": null,
"genotype": [
1,
1
],
"genotype_likelihoods": [
-4404.478230939082,
-99999999,
-40.687503862340144
],
"info": {
"coverage": {
"reference": {
"percent_coverage": 0.0,
"median_depth": 0,
"min_non_zero_depth": 0,
"kmer_count": 0,
"klen": 21
},
"alternate": {
"percent_coverage": 100.0,
"median_depth": 35,
"min_non_zero_depth": 31,
"kmer_count": 674,
"klen": 20
}
},
"expected_depths": [
39
],
"contamination_depths": [],
"filter": [],
"conf": 4364
},
"_cls": "Call.VariantCall
}
}
}
Amikacin": {
"predict": "S

}
Capreomycin": {
"predict": "r

called_by": {
"rrs_C1402X-C1473247A": {
"variant": null,
"genotype": [
0,
1
],
"genotype_likelihoods": [
-1899.0356937696108,
-778.6609519775338,
-5231.24598764316
],
"info": {
"coverage": {
"reference": {
"percent_coverage": 100.0,
"median_depth": 42,
"min_non_zero_depth": 39,
"kmer_count": 1416,
"klen": 21
},
"alternate": {
"percent_coverage": 100.0,
"median_depth": 31,
"min_non_zero_depth": 23,
"kmer_count": 602,
"klen": 21
}
},
"expected_depths": [
39
],
"contamination_depths": [],
"filter": [],
"conf": 1120
},
"_cls": "Call.VariantCall
}
}

}
}

phylogenetics": {
"phylo_group": {
"Mycobacterium_tuberculosis_complex": {
"percent_coverage": 99.564,
"median_depth": 39
}
},
"sub_complex": {
"Unknown": {
"percent_coverage": -1,
"median_depth": -1
}
},
"species": {
"Mycobacterium_tuberculosis": {
"percent_coverage": 98.514,
"median_depth": 34
}
},
"lineage": {
"lineage": [
"lineage2.2
]

calls_summary": {
"lineage2.2": {
"good_nodes": 2,
"tree_depth": 2,
"genotypes": {
"lineage2": 1,
"lineage2.2": 1
}
}
},
"calls": {
"lineage2.2": {
"lineage2": {
"G497491A": {
"variant": "ref-G497491A?var_name=G497491A&num_alts=1&ref=NC_000962.3&enum=0&gene=NA&mut=G497491A

genotype": [
1,
1
],
"genotype_likelihoods": [
-4601.386515514585,
-99999999,
-32.50342569218019
],
"info": {
"coverage": {
"reference": {
"percent_coverage": 0.0,
"median_depth": 0,
"min_non_zero_depth": 0,
"kmer_count": 0,
"klen": 21
},
"alternate": {
"percent_coverage": 100.0,
"median_depth": 39,
"min_non_zero_depth": 25,
"kmer_count": 732,
"klen": 21
}
},
"expected_depths": [
39
],
"contamination_depths": [],
"filter": [],
"conf": 4569
},
"_cls": "Call.VariantCall
}
}
lineage2.2": {
"G2505085A": {
"variant": "ref-G2505085A?var_name=G2505085A&num_alts=2&ref=NC_000962.3&enum=0&gene=NA&mut=G2505085A

```
genotype": [
                        1,
                        1
                    ],
                    "genotype_likelihoods": [
                        -4423.608355834194,
                        -99999999,
                        -38.93836579595363
                    ],
                    "info": {
                        "coverage": {
                            "reference": {
                                "percent_coverage": 0.0,
                                "median_depth": 0,
                                "min_non_zero_depth": 0,
                                "kmer_count": 0,
                                "klen": 21
                            },
                            "alternate": {
                                "percent_coverage": 100.0,
                                "median_depth": 34,
                                "min_non_zero_depth": 32,
                                "kmer_count": 687,
                                "klen": 21
                            }
                        },
                        "expected_depths": [
                            39
                        ],
                        "contamination_depths": [],
                        "filter": [],
                        "conf": 4385
                    },
                    "_cls": "Call.VariantCall
                }
            }
        }
    }
}
}
```

kmer": 21,
"probe_sets": [
"/home/idowu/miniconda3/lib/python3.6/site-packages/mykrobe/data/tb/tb-species-170421.fasta.gz
/home/idowu/miniconda3/lib/python3.6/site-packages/mykrobe/data/tb/tb-hunt-probe-set-jan-03-2019.fasta.gz
/home/idowu/miniconda3/lib/python3.6/site-packages/mykrobe/data/tb/tb.lineage.20200930.probes.fa.gz
]
files": [
"TB3qc_R1.fastq.gz
TB3qc_R2.fastq.gz
]
version": {
"mykrobe-predictor": "v0.9.0
mykrobe-atlas": "v0.9.0
}
genotype_model": "kmer_count
}
}
