# Supplementary material for: Whole genome sequencing of clinical samples reveals extensively drug resistant tuberculosis (XDR TB) strains from the Beijing lineage in Nigeria, West Africa
Source: Sci Rep. 2021 Aug 30;11:17387. doi: 10.1038/s41598-021-96956-7 (PMC8405707; doi:10.1038/s41598-021-96956-7)
Supplement: Supplementary file 3 — Supplementary Information 3. [file 41598_2021_96956_MOESM3_ESM.pdf]

TBProfiler report

Summary

ID	TB3_tbprofiler
Date	Mon May 3 15:43:30 2021
Strain	lineage2.2.1
Drug-resistance	XDR

Lineage report

Lineage	Estimated Fraction	Family	Spoligotype	Rd
lineage2	0.983	East-Asian	Beijing	RD105
lineage2.2	1	East-Asian (Beijing)	Beijing-RD207	RD105;RD207
lineage2.2.1	1	East-Asian (Beijing)	Beijing-RD181	RD105;RD207;RD181

Resistance report

Drug	Genotypic Resistance	Mutations
Rifampicin	R	rpoB p.Ser450Leu (1.00)
Isoniazid	R	katG c.1561 1561del (0.12)
Ethambutol	R	embB p.Gln497Arg (1.00)
Pyrazinamide	R	pncA p.Asp12Gly (1.00)
Streptomycin	R	rpsL p.Lys43Arg (0.91), rrs r.799c>t (0.16)
Fluoroquinolones	R	gyrA p.Ala90Val (1.00), gyrA p.Ser91Pro (1.00)
Amikacin	R	rrs r.1402c>a (0.39), rrs r.1484g>t (0.14)
Capreomycin	R	rrs r.1402c>a (0.39), rrs r.1484g>t (0.14)
Kanamycin	R	rrs r.1402c>a (0.39), rrs r.1484g>t (0.14)
Cycloserine		
Ethionamide		
Clofazimine		
Para-aminosalicylic acid		

Delamanid
Bedaquiline
Linezolid

Resistance variants report

Genome Position	Locus Tag	Gene	Change	Estimated Fraction
7570	Rv0006	gyrA	p.Ala90Val	1
7572	Rv0006	gyrA	p.Ser91Pro	1
761155	Rv0667	rpoB	p.Ser450Leu	1
781687	Rv0682	rpsL	p.Lys43Arg	0.909
1472644	rrs	rrs	r.799c>t	0.16
1473247	rrs	rrs	r.1402c>a	0.389
1473329	rrs	rrs	r.1484g>t	0.143
2154550	Rv1908c	katG	c.1561 1561del	0.115
2289207	Rv2043c	pncA	p.Asp12Gly	1
4248003	Rv3795	embB	p.Gln497Arg	1

Other variants report

Genome Position	Locus Tag	Change	Estimated Fraction
5726	Rv0005	p.Pro163Thr	0.118
5819	Rv0005	p.Leu194Met	0.091
5968	Rv0005	c.729G>A	0.167
6051	Rv0005	p.Ile271Thr	0.118
6377	Rv0005	p.Val380Ile	0.143
6534	Rv0005	p.Ala432Asp	0.143
7362	Rv0006	p.Glu21Gln	1
7484	Rv0006	c.183T>C	0.143
7585	Rv0006	p.Ser95Thr	1
7604	Rv0006	c.303 304insC	0.111
7641	Rv0006	p.Gly114Cys	0.1
8777	Rv0006	p.Asp492Glu	0.154

9117	Rv0006	p.Glu606Tyr	0.1
9641	Rv0006	c.2340C>T	0.087
490748	Rv0407	c.-35G>T	0.091
490753	Rv0407	c.-30G>T	0.136
490949	Rv0407	p.Ser56Tyr	0.154
491011	Rv0407	p.Pro77Thr	0.105
491256	Rv0407	p.Lys158Asn	0.2
491664	Rv0407	c.882C>A	0.125
491717	Rv0407	p.Ala312Glu	0.118
491742	Rv0407	c.960T>C	1
575659	Rv0486	c.175 176insC	0.167
575907	Rv0486	p.Ala187Val	1
576315	Rv0486	p.Ala323Val	0.167
576784	Rv0486	c.1300C>A	0.167
759457	Rv0667	c.-350C>A	0.111
760491	Rv0667	p.Thr229Ala	0.2
760760	Rv0667	c.954C>T	0.182
761012	Rv0667	p.Asp402Glu	0.154
761166	Rv0667	p.Pro454Ala	0.154
761234	Rv0667	c.1428G>C	0.182
761255	Rv0667	c.1449T>G	0.2
761258	Rv0667	c.1452G>A	0.167
761261	Rv0667	c.1455G>C	0.154
761264	Rv0667	c.1458C>G	0.154
761273	Rv0667	c.1467T>C	0.182
761282	Rv0667	c.1476C>G	0.2
761777	Rv0667	p.Asp657Glu	0.143
762081	Rv0667	p.Gly759Cys	0.087
762362	Rv0667	p.Glu852Asp	0.125
762815	Rv0667	p.Met1003Ile	0.2
762842	Rv0667	c.3036G>T	0.182
762951	Rv0667	p.Gln1049Lys	0.167
763031	Rv0667	c.3225T>C	1
763037	Rv0667	p.Tyr1077*	0.111
763083	Rv0667	p.Arg1093Ser	0.182

763570	Rv0668	c.201G>C	0.091
763772	Rv0668	p.Val135Leu	0.133
763934	Rv0668	p.Ala189Ser	0.105
764229	Rv0668	p.Gln287Arg	0.2
764371	Rv0668	c.1002G>C	0.182
764575	Rv0668	c.1206T>G	0.2
764605	Rv0668	c.1236G>C	0.167
764611	Rv0668	c.1242G>T	0.235
764632	Rv0668	c.1263T>C	0.19
764635	Rv0668	c.1266C>G	0.136
764650	Rv0668	c.1281G>C	0.235
764665	Rv0668	c.1296C>G	0.267
764668	Rv0668	c.1299C>T	0.267
764671	Rv0668	c.1302G>C	0.286
764672	Rv0668	p.Gln435Lys	0.133
764677	Rv0668	c.1308C>G	0.133
764695	Rv0668	c.1326T>C	0.333
764701	Rv0668	c.1332C>G	0.231
764706	Rv0668	p.Leu446Gln	0.417
764746	Rv0668	c.1377G>T	0.2
764749	Rv0668	c.1380G>C	0.188
764758	Rv0668	c.1389C>G	0.118
764764	Rv0668	c.1395T>C	0.125
764828	Rv0668	p.Leu487Ile	0.1
764854	Rv0668	c.1485G>T	0.105
764916	Rv0668	p.Leu516Pro	0.947
765722	Rv0668	p.Val785Ile	0.167
765891	Rv0668	p.Arg841Leu	0.133
766310	Rv0668	p.Arg981Ser	0.2
767150	Rv0668	p.Gly1261Ser	0.091
767212	Rv0668	c.3843G>T	0.105
779223	Rv0678	c.234G>T	0.105
779230	Rv0678	p.Glu81*	0.091
781395	Rv0682	c.-165T>C	1
801041	Rv0701	p.Arg78Leu	0.133

801103	Rv0701	p.Gln99Lys	0.167
801141	Rv0701	p.Tyr111*	0.154
1302694	Rv1173	c.-237C>A	0.105
1302776	Rv1173	c.-155C>A	0.125
1304097	Rv1173	c.1167G>A	0.167
1416573	Rv1267c	p.Pro259Thr	0.143
1416853	Rv1267c	c.495C>T	0.111
1461167	Rv1305	c.123G>T	0.143
1472108	rrs	r.263c>t	0.208
1472112	rrs	r.267c>t	0.091
1472113	rrs	r.268t>c	0.087
1472122	rrs	r.277g>a	0.1
1472137	rrs	r.292g>a	0.118
1472148	rrs	r.303t>c	0.182
1472150	rrs	r.305t>a	0.619
1472151	rrs	r.306c>t	0.095
1472172	rrs	r.327t>c	0.545
1472214	rrs	r.369c>g	0.259
1472235	rrs	r.390g>c	0.24
1472240	rrs	r.395g>c	0.111
1472251	rrs	r.406g>a	0.286
1472286	rrs	r.441c>g	0.125
1472289	rrs	r.444t>g	0.167
1472290	rrs	r.445c>g	0.167
1472297	rrs	r.452ggtccgggttctct>g	0.2
1472324	rrs	r.479g>c	0.188
1472325	rrs	r.480g>c	0.118
1472327	rrs	r.482g>a	0.118
1472328	rrs	r.483g>c	0.118
1472330	rrs	r.485g>t	0.105
1472344	rrs	r.499c>t	0.333
1472382	rrs	r.537g>a	0.12
1472389	rrs	r.544g>a	0.154
1472400	rrs	r.555c>t	0.174
1472422	rrs	r.577t>c	0.1

1472430	rrs	r.585c>t	0.1
1472489	rrs	r.644a>t	0.107
1472496	rrs	r.651t>g	0.087
1472655	rrs	r.810g>t	0.125
1472660	rrs	r.815t>c	0.125
1472673	rrs	r.828t>g	0.167
1472674	rrs	r.829t>a	0.133
1472675	rrs	r.830t>c	0.133
1472677	rrs	r.832c>a	0.188
1472682	rrs	r.837t>a	0.143
1472683	rrs	r.838t>c	0.154
1472686	rrs	r.841g>t	0.154
1472687	rrs	r.842a>t	0.154
1472692	rrs	r.847t>c	0.214
1472697	rrs	r.852t>c	0.125
1472707	rrs	r.862a>t	0.158
1472714	rrs	r.869a>g	0.158
1472715	rrs	r.870c>g	0.105
1472716	rrs	r.871c>a	0.105
1472734	rrs	r.889c>t	0.263
1472740	rrs	r.895g>t	0.1
1472741	rrs	r.896g>a	0.35
1472742	rrs	r.897c>t	0.3
1472754	rrs	r.909g>t	0.304
1472767	rrs	r.922g>c	0.133
1472781	rrs	r.936c>t	0.31
1472790	rrs	r.945t>c	0.095
1472793	rrs	r.948a>t	0.341
1472803	rrs	r.958t>c	0.189
1472828	rrs	r.983t>c	0.143
1472836	rrs	r.991g>a	0.095
1472952	rrs	r.1107t>c	0.115
1472973	rrs	r.1128a>t	0.286
1472974	rrs	r.1129a>g	0.088
1472987	rrs	r.1142g>a	0.304

1472990	rrs	r.1145a>g	0.28
1472996	rrs	r.1151t>c	0.219
1473026	rrs	r.1181t>c	0.103
1473035	rrs	r.1190g>a	0.179
1473055	rrs	r.1210c>t	0.172
1473056	rrs	r.1211a>t	0.12
1473062	rrs	r.1217t>g	0.091
1473065	rrs	r.1220c>a	0.087
1473066	rrs	r.1221a>g	0.304
1473081	rrs	r.1236c>t	0.133
1473088	rrs	r.1243a>g	0.111
1473099	rrs	r.1254t>g	0.087
1473100	rrs	r.1255g>a	0.136
1473102	rrs	r.1257c>t	0.087
1473104	rrs	r.1259c>t	0.174
1473110	rrs	r.1265t>g	0.107
1473111	rrs	r.1266a>g	0.143
1473121	rrs	r.1276t>c	0.167
1473145	rrs	r.1300c>t	0.222
1473166	rrs	r.1321g>a	0.292
1473177	rrs	r.1332g>a	0.087
1473192	rrs	r.1347a>g	0.105
1473198	rrs	r.1353gc>g	0.125
1473202	rrs	r.1357c>t	0.125
1473205	rrs	r.1360t>c	0.125
1473226	rrs	r.1381c>g	0.214
1473248	rrs	r.1403g>a	0.118
1473249	rrs	r.1404t>c	0.111
1473252	rrs	r.1407t>c	0.333
1473259	rrs	r.1414c>t	0.286
1473276	rrs	r.1431a>g	0.471
1473283	rrs	r.1438t>c	0.211
1473288	rrs	r.1443c>g	0.111
1473290	rrs	r.1445c>t	0.333
1473301	rrs	r.1456t>c	0.389

1473315	rrs	r.1470t>c	0.214
1473316	rrs	r.1471c>t	0.214
1473319	rrs	r.1474c>t	0.143
1473352	rrs	r.1507c>t	0.385
1473363	rrs	r.1518ggc>g	0.154
1474161	rrl	r.504a>ac	0.105
1474184	rrl	r.527c>t	0.094
1474249	rrl	r.592g>t	0.25
1474263	rrl	r.606g>a	0.158
1474275	rrl	r.618t>a	0.105
1474498	rrl	r.841g>t	0.111
1474505	rrl	r.848c>g	0.1
1474516	rrl	r.859c>a	0.095
1474529	rrl	r.872a>c	0.158
1474530	rrl	r.873g>a	0.143
1474537	rrl	r.880g>a	0.13
1474539	rrl	r.882c>t	0.125
1474540	rrl	r.883t>g	0.143
1474549	rrl	r.892g>t	0.105
1474644	rrl	r.987g>a	0.111
1474736	rrl	r.1079c>t	0.133
1474749	rrl	r.1092c>t	0.286
1474751	rrl	r.1094g>a	0.154
1474753	rrl	r.1096ac>c	0.2
1474760	rrl	r.1103a>g	0.474
1474770	rrl	r.1113g>a	0.143
1474777	rrl	r.1120t>c	0.273
1474779	rrl	r.1122g>a	0.455
1474780	rrl	r.1123c>t	0.091
1474782	rrl	r.1125g>a	0.227
1474784	rrl	r.1127c>t	0.208
1474790	rrl	r.1133c>t	0.125
1474794	rrl	r.1137c>t	0.417
1474799	rrl	r.1142gt>g	0.1
1474823	rrl	r.1166c>g	0.364

1474827 rrl	r.1170c>t	0.364
1474830 rrl	r.1173a>t	0.25
1474831 rrl	r.1174a>g	0.222
1474904 rrl	r.1247g>c	0.382
1474905 rrl	r.1248t>c	0.406
1474913 rrl	r.1256t>c	0.323
1474932 rrl	r.1275c>t	0.235
1475158 rrl	r.1501c>t	0.125
1475645 rrl	r.1988c>a	0.125
1475699 rrl	r.2042c>t	0.136
1475722 rrl	r.2065g>t	0.222
1475753 rrl	r.2096c>t	0.167
1475765 rrl	r.2108a>t	0.167
1475769 rrl	r.2112t>c	0.143
1475781 rrl	r.2124t>c	0.2
1475791 rrl	r.2134a>g	0.1
1475803 rrl	r.2146t>c	0.095
1475804 rrl	r.2147g>c	0.095
1475816 rrl	r.2159c>g	0.214
1475817 rrl	r.2160a>g	0.267
1475869 rrl	r.2212c>a	0.213
1475877 rrl	r.2220c>t	0.195
1475881 rrl	r.2224t>c	0.25
1475883 rrl	r.2226a>t	0.256
1475884 rrl	r.2227a>g	0.156
1475892 rrl	r.2235a>c	0.188
1475898 rrl	r.2241a>g	0.239
1475899 rrl	r.2242g>a	0.239
1475906 rrl	r.2249c>t	0.216
1475916 rrl	r.2259c>t	0.212
1475943 rrl	r.2286g>a	0.208
1475952 rrl	r.2295a>g	0.175
1475970 rrl	r.2313c>t	0.204
1475975 rrl	r.2318c>t	0.318
1475977 rrl	r.2320a>g	0.359

1475978 rrl	r.2321c>t	0.231
1475988 rrl	r.2331a>g	0.25
1475990 rrl	r.2333g>a	0.091
1475991 rrl	r.2334t>a	0.091
1475993 rrl	r.2336c>t	0.111
1476000 rrl	r.2343g>a	0.105
1476260 rrl	r.2603a>g	0.174
1476294 rrl	r.2637a>g	0.091
1476295 rrl	r.2638c>g	0.091
1476296 rrl	r.2639c>t	0.091
1476297 rrl	r.2640c>t	0.087
1476302 rrl	r.2645g>a	0.087
1476332 rrl	r.2675g>c	0.105
1476336 rrl	r.2679c>t	0.175
1476338 rrl	r.2681c>t	0.108
1476353 rrl	r.2696g>t	0.103
1476356 rrl	r.2699c>a	0.225
1476357 rrl	r.2700t>c	0.231
1476358 rrl	r.2701t>c	0.35
1476359 rrl	r.2702c>g	0.39
1476369 rrl	r.2712c>t	0.41
1476372 rrl	r.2715t>c	0.154
1476381 rrl	r.2724g>c	0.469
1476382 rrl	r.2725a>g	0.424
1476383 rrl	r.2726t>g	0.296
1476384 rrl	r.2727g>t	0.235
1476408 rrl	r.2751g>a	0.343
1476411 rrl	r.2754g>a	0.161
1476425 rrl	r.2768g>a	0.353
1476428 rrl	r.2771c>t	0.571
1476429 rrl	r.2772a>c	0.389
1476442 rrl	r.2785t>a	0.184
1476443 rrl	r.2786g>t	0.189
1476455 rrl	r.2798c>a	0.175
1476456 rrl	r.2799a>t	0.163

1476463	rrl	r.2806c>a	0.154
1476466	rrl	r.2809c>t	0.537
1476481	rrl	r.2824t>c	0.4
1476506	rrl	r.2849t>c	0.571
1476513	rrl	r.2856g>t	0.1
1476514	rrl	r.2857c>t	0.105
1476515	rrl	r.2858c>t	0.118
1476524	rrl	r.2867c>t	0.143
1476525	rrl	r.2868a>g	0.2
1476530	rrl	r.2873c>t	0.143
1476536	rrl	r.2879g>a	0.143
1476538	rrl	r.2881a>g	0.143
1476547	rrl	r.2890c>t	0.375
1476565	rrl	r.2908g>a	0.125
1476566	rrl	r.2909a>g	0.125
1476567	rrl	r.2910c>t	0.312
1476573	rrl	r.2916a>c	0.118
1476577	rrl	r.2920t>g	0.118
1476584	rrl	r.2927c>t	0.312
1476588	rrl	r.2931a>g	0.176
1673524	Rv1483	c.85C>T	0.143
1674892	Rv1484	p.Asn231Asp	1
1834015	Rv1630	c.474G>C	0.143
1834040	Rv1630	p.Lys167Gln	0.154
1834135	Rv1630	c.594G>C	0.2
1834177	Rv1630	c.636A>C	1
2153993	Rv1908c	p.Leu707Ile	0.154
2154003	Rv1908c	p.Glu703Asp	0.125
2154057	Rv1908c	c.2055G>T	0.154
2155396	Rv1908c	p.Pro239Leu	0.154
2288710	Rv2043c	p.Ala178Pro	0.143
2288745	Rv2043c	p.Asp166Gly	0.125
2518264	Rv2245	c.150C>T	0.125
2518323	Rv2245	p.Ser70Asn	0.091
2518433	Rv2245	p.Arg107Trp	0.182

2518849	Rv2245	c.735G>T	0.1
2519053	Rv2245	c.939G>C	0.118
2714138	Rv2416c	p.Ala399Pro	0.133
2714317	Rv2416c	p.Arg339Gln	0.091
2714377	Rv2416c	p.Thr319Lys	0.091
2726152	Rv2428	c.-41C>A	0.136
2746755	Rv2447c	p.Leu282Ile	0.125
2747136	Rv2447c	p.Leu155Ile	0.167
2859519	Rv2535c	c.900C>T	0.154
2986852	Rv2671	p.Gly5Val	0.111
2987361	Rv2671	p.Glu175*	0.125
3067716	Rv2754c	p.Ser77Ile	0.125
3067743	Rv2754c	p.Gly68Val	0.154
3068076	Rv2754c	c.-131G>T	0.1
3073905	Rv2764c	c.567G>A	0.125
3074096	Rv2764c	c.376G>T	0.167
3074116	Rv2764c	p.Leu119Pro	0.143
3074150	Rv2764c	p.His108Asn	0.111
3074446	Rv2764c	p.Arg9His	0.118
3086788	Rv2780	c.-32T>C	1
3086950	Rv2780	p.Ala44Gly	0.125
3086977	Rv2780	p.Ala53Glu	0.118
3087294	Rv2780	p.Leu159Met	0.133
3087419	Rv2780	p.Asn200Lys	0.118
3087786	Rv2780	p.Glu323*	0.091
3339237	Rv2983	c.120G>T	0.105
3641545	Rv3262	p.Pro4Leu	0.133
3642540	Rv3262	p.Ala336Pro	0.087
3840248	Rv3423c	c.1173G>T	0.105
3840555	Rv3423c	p.Ala289Val	0.1
3840890	Rv3423c	c.531G>A	0.111
3840913	Rv3423c	p.Ala170Thr	0.143
3841546	Rv3423c	c.-126C>A	0.212
3841662	Rv3423c	c.-242A>G	0.313
3841677	Rv3423c	c.-257C>T	0.197

3841679	Rv3423c	c.-259T>G	0.177
3841694	Rv3423c	c.-274C>A	0.231
3987026	Rv3547	c.183C>A	0.091
4241072	Rv3793	p.Leu404Met	0.182
4241439	Rv3793	p.Ser526Asn	0.111
4241733	Rv3793	p.Ala624Val	0.143
4241864	Rv3793	c.2002C>T	0.143
4242900	Rv3793	p.Pro1013Arg	0.111
4242967	Rv3793	p.Ile1035Met	0.105
4243318	Rv3794	p.Gly29Asp	0.167
4244354	Rv3794	c.1122G>A	0.1
4244850	Rv3794	p.Arg540Trp	0.2
4244891	Rv3794	c.1659C>T	0.2
4245757	Rv3794	p.Ser842Ile	0.167
4246176	Rv3794	p.Ala982Thr	0.2
4246544	Rv3795	p.Thr11Pro	0.6
4247387	Rv3795	p.Val292Phe	0.091
4247470	Rv3795	c.957T>C	0.095
4247472	Rv3795	p.Phe320Tyr	0.095
4248683	Rv3795	c.2170 2171insAT	0.105
4249676	Rv3795	p.Arg1055Gly	0.2
4249757	Rv3795	p.Thr1082Ala	1
4269437	Rv3806c	p.Ala133Ser	0.1
4326405	Rv3854c	p.Asp357Tyr	1
4326970	Rv3854c	c.504G>T	0.087
4327430	Rv3854c	p.Ser15Tyr	0.111
4327701	Rv3855	c.153C>A	0.13
4327731	Rv3855	p.Phe61Leu	0.087
4327933	Rv3855	p.Ala129Thr	0.133
4407588	Rv3919c	c.615T>C	1
4407927	Rv3919c	p.Glu92Asp	1
4408085	Rv3919c	p.Glu40*	0.133
4408200	Rv3919c	3C>A	0.1

Coverage report

Gene	Locus_Tag	Cutoff	Fraction
gyrB	Rv0005	0	0
gyrA	Rv0006	0	0
fgd1	Rv0407	0	0
mshA	Rv0486	0	0
rpoB	Rv0667	0	0
rpoC	Rv0668	0	0
mmpR5	Rv0678	0	0
rpsL	Rv0682	0	0
rplC	Rv0701	0	0
fbiC	Rv1173	0	0
embR	Rv1267c	0	0
atpE	Rv1305	0	0
rrs	rrs	0	0
rrl	rrl	0	0
fabG1	Rv1483	0	0
inhA	Rv1484	0	0
rpsA	Rv1630	0	0
tlyA	Rv1694	0	0
katG	Rv1908c	0	0
pncA	Rv2043c	0	0
kasA	Rv2245	0	0
eis	Rv2416c	0	0
ahpC	Rv2428	0	0
folC	Rv2447c	0	0
pepQ	Rv2535c	0	0
ribD	Rv2671	0	0
thyX	Rv2754c	0	0
thyA	Rv2764c	0	0
ald	Rv2780	0	0
fbiD	Rv2983	0	0
fbiA	Rv3261	0	0
fbiB	Rv3262	0	0

alr	Rv3423c	0	0
ddn	Rv3547	0	0
panD	Rv3601c	0	0
embC	Rv3793	0	0
embA	Rv3794	0	0
embB	Rv3795	0	0
ubiA	Rv3806c	0	0
ethA	Rv3854c	0	0
ethR	Rv3855	0	0
gid	Rv3919c	0	0

Missing positions report

Gene	Locus_Tag	Position	Position_Type	Drug_Resistance_Positio
fgd1	Rv0407	186	codon	
fgd1	Rv0407	187	codon	
fgd1	Rv0407	188	codon	
fgd1	Rv0407	189	codon	
fgd1	Rv0407	190	codon	
fgd1	Rv0407	191	codon	
fgd1	Rv0407	192	codon	
fgd1	Rv0407	193	codon	
fgd1	Rv0407	194	codon	
fgd1	Rv0407	195	codon	
fgd1	Rv0407	196	codon	
fgd1	Rv0407	197	codon	
fgd1	Rv0407	198	codon	
fgd1	Rv0407	199	codon	
fgd1	Rv0407	200	codon	
fgd1	Rv0407	201	codon	
fgd1	Rv0407	202	codon	
fgd1	Rv0407	203	codon	
fgd1	Rv0407	204	codon	
fgd1	Rv0407	205	codon	

fgd1	Rv0407	207	codon
fgd1	Rv0407	208	codon
fgd1	Rv0407	209	codon
fgd1	Rv0407	221	codon
fgd1	Rv0407	222	codon
fgd1	Rv0407	223	codon
fgd1	Rv0407	224	codon
fgd1	Rv0407	225	codon
mshA	Rv0486	308	codon
mshA	Rv0486	309	codon
mshA	Rv0486	310	codon
mshA	Rv0486	319	codon
mshA	Rv0486	320	codon
mshA	Rv0486	321	codon
mshA	Rv0486	322	codon
mshA	Rv0486	323	codon
mshA	Rv0486	324	codon
mshA	Rv0486	325	codon
mshA	Rv0486	326	codon
mshA	Rv0486	327	codon
mshA	Rv0486	328	codon
mshA	Rv0486	329	codon
mshA	Rv0486	330	codon
mshA	Rv0486	331	codon
mshA	Rv0486	332	codon
mshA	Rv0486	333	codon
mshA	Rv0486	334	codon
mshA	Rv0486	335	codon
mshA	Rv0486	336	codon
mshA	Rv0486	337	codon
mshA	Rv0486	338	codon
mshA	Rv0486	339	codon
mshA	Rv0486	381	codon
mshA	Rv0486	382	codon
mshA	Rv0486	383	codon

mshA	Rv0486	384 codon
mshA	Rv0486	385 codon
mshA	Rv0486	386 codon
mshA	Rv0486	387 codon
mshA	Rv0486	388 codon
rpoB	Rv0667	244 codon
rpoB	Rv0667	245 codon
rpoB	Rv0667	246 codon
rpoB	Rv0667	247 codon
rpoB	Rv0667	248 codon
rpoB	Rv0667	262 codon
rpoB	Rv0667	263 codon
rpoB	Rv0667	264 codon
rpoB	Rv0667	623 codon
rpoB	Rv0667	624 codon
rpoB	Rv0667	625 codon
rpoB	Rv0667	626 codon
rpoB	Rv0667	627 codon
rpoB	Rv0667	628 codon
rpoB	Rv0667	989 codon
rpoB	Rv0667	990 codon
rpoB	Rv0667	991 codon
rpoC	Rv0668	263 codon
rpoC	Rv0668	264 codon
rpoC	Rv0668	265 codon
rpoC	Rv0668	266 codon
rpoC	Rv0668	267 codon
rpoC	Rv0668	268 codon
rpoC	Rv0668	269 codon
rpoC	Rv0668	270 codon
rpoC	Rv0668	271 codon
rpoC	Rv0668	272 codon
rpoC	Rv0668	273 codon
rpoC	Rv0668	274 codon
rpoC	Rv0668	275 codon

rpoC	Rv0668	276	codon
rpoC	Rv0668	791	codon
rpoC	Rv0668	792	codon
rpoC	Rv0668	793	codon
rpoC	Rv0668	794	codon
rpoC	Rv0668	1202	codon
rpoC	Rv0668	1203	codon
rpoC	Rv0668	1204	codon
rpoC	Rv0668	1205	codon
rpoC	Rv0668	1206	codon
rpoC	Rv0668	1207	codon
rpoC	Rv0668	1208	codon
rpoC	Rv0668	1209	codon
rpoC	Rv0668	1210	codon
rpoC	Rv0668	1211	codon
rpoC	Rv0668	1212	codon
rpoC	Rv0668	1213	codon
rpoC	Rv0668	1214	codon
rpoC	Rv0668	1215	codon
rpoC	Rv0668	1216	codon
rpoC	Rv0668	1217	codon
rpoC	Rv0668	1218	codon
rpoC	Rv0668	1219	codon
rpoC	Rv0668	1220	codon
rpoC	Rv0668	1221	codon
rpoC	Rv0668	1222	codon
rpoC	Rv0668	1223	codon
rpoC	Rv0668	1224	codon
rpoC	Rv0668	1225	codon
rpsA	Rv1630	358	codon
rpsA	Rv1630	359	codon
rpsA	Rv1630	360	codon
rpsA	Rv1630	361	codon
rpsA	Rv1630	362	codon
rpsA	Rv1630	363	codon

rpsA	Rv1630	364	codon
rpsA	Rv1630	365	codon
rpsA	Rv1630	366	codon
rpsA	Rv1630	367	codon
rpsA	Rv1630	368	codon
rpsA	Rv1630	385	codon
rpsA	Rv1630	386	codon
rpsA	Rv1630	387	codon
rpsA	Rv1630	388	codon
rpsA	Rv1630	389	codon
rpsA	Rv1630	390	codon
rpsA	Rv1630	391	codon
rpsA	Rv1630	392	codon
rpsA	Rv1630	393	codon
rpsA	Rv1630	394	codon
rpsA	Rv1630	395	codon
rpsA	Rv1630	396	codon
rpsA	Rv1630	397	codon
rpsA	Rv1630	398	codon
rpsA	Rv1630	399	codon
rpsA	Rv1630	400	codon
rpsA	Rv1630	401	codon
rpsA	Rv1630	402	codon
rpsA	Rv1630	403	codon
rpsA	Rv1630	463	codon
rpsA	Rv1630	464	codon
rpsA	Rv1630	465	codon
rpsA	Rv1630	466	codon
rpsA	Rv1630	467	codon
rpsA	Rv1630	468	codon
rpsA	Rv1630	469	codon
rpsA	Rv1630	470	codon
rpsA	Rv1630	471	codon
rpsA	Rv1630	472	codon
rpsA	Rv1630	473	codon

rpsA	Rv1630	474 codon	pyrazinamide
rpsA	Rv1630	475 codon	
rpsA	Rv1630	476 codon	
rpsA	Rv1630	477 codon	
rpsA	Rv1630	478 codon	
katG	Rv1908c	265 codon	isoniazid
katG	Rv1908c	264 codon	isoniazid
katG	Rv1908c	263 codon	
katG	Rv1908c	262 codon	isoniazid
katG	Rv1908c	261 codon	isoniazid
katG	Rv1908c	260 codon	
katG	Rv1908c	259 codon	isoniazid
katG	Rv1908c	116 codon	
kasA	Rv2245	164 codon	
kasA	Rv2245	165 codon	
kasA	Rv2245	166 codon	
kasA	Rv2245	167 codon	
kasA	Rv2245	168 codon	
kasA	Rv2245	169 codon	
kasA	Rv2245	170 codon	
kasA	Rv2245	171 codon	
folC	Rv2447c	353 codon	
pepQ	Rv2535c	266 codon	
pepQ	Rv2535c	265 codon	
pepQ	Rv2535c	264 codon	
pepQ	Rv2535c	263 codon	
pepQ	Rv2535c	262 codon	
pepQ	Rv2535c	261 codon	
pepQ	Rv2535c	260 codon	
pepQ	Rv2535c	259 codon	
pepQ	Rv2535c	258 codon	
pepQ	Rv2535c	249 codon	
pepQ	Rv2535c	248 codon	
pepQ	Rv2535c	247 codon	
pepQ	Rv2535c	246 codon	

pepQ	Rv2535c	245	codon
pepQ	Rv2535c	244	codon
pepQ	Rv2535c	243	codon
pepQ	Rv2535c	242	codon
pepQ	Rv2535c	241	codon
pepQ	Rv2535c	214	codon
pepQ	Rv2535c	211	codon
pepQ	Rv2535c	210	codon
pepQ	Rv2535c	209	codon
pepQ	Rv2535c	208	codon
pepQ	Rv2535c	207	codon
pepQ	Rv2535c	206	codon
ribD	Rv2671	90	codon
ribD	Rv2671	91	codon
ribD	Rv2671	92	codon
ribD	Rv2671	93	codon
ribD	Rv2671	94	codon
ribD	Rv2671	95	codon
ribD	Rv2671	96	codon
thyX	Rv2754c	222	codon
thyX	Rv2754c	221	codon
thyX	Rv2754c	220	codon
thyX	Rv2754c	219	codon
thyX	Rv2754c	218	codon
thyX	Rv2754c	217	codon
thyX	Rv2754c	216	codon
thyX	Rv2754c	215	codon
thyX	Rv2754c	214	codon
thyX	Rv2754c	213	codon
thyX	Rv2754c	212	codon
thyX	Rv2754c	211	codon
thyX	Rv2754c	210	codon
thyX	Rv2754c	209	codon
thyX	Rv2754c	208	codon
thyX	Rv2754c	207	codon

thyX	Rv2754c	206	codon	
thyX	Rv2754c	205	codon	
thyX	Rv2754c	204	codon	
thyX	Rv2754c	203	codon	
alr	Rv3423c	86	codon	
alr	Rv3423c	85	codon	
embC	Rv3793	427	codon	
embC	Rv3793	428	codon	
embC	Rv3793	429	codon	
embC	Rv3793	430	codon	
embC	Rv3793	431	codon	
embC	Rv3793	432	codon	
embC	Rv3793	450	codon	
embC	Rv3793	451	codon	
embC	Rv3793	452	codon	
embC	Rv3793	453	codon	
embC	Rv3793	456	codon	
embC	Rv3793	457	codon	
embC	Rv3793	779	codon	ethambutol
embC	Rv3793	780	codon	
embC	Rv3793	781	codon	
embC	Rv3793	794	codon	
embC	Rv3793	795	codon	
embC	Rv3793	796	codon	
embC	Rv3793	797	codon	
embC	Rv3793	798	codon	
embC	Rv3793	801	codon	
embC	Rv3793	802	codon	
embC	Rv3793	803	codon	
embC	Rv3793	804	codon	
embC	Rv3793	805	codon	
embC	Rv3793	806	codon	
embC	Rv3793	807	codon	
embC	Rv3793	808	codon	
embC	Rv3793	809	codon	

embC	Rv3793	810	codon	
embC	Rv3793	811	codon	
embC	Rv3793	812	codon	
embC	Rv3793	813	codon	
embC	Rv3793	814	codon	
embA	Rv3794	543	codon	
embA	Rv3794	544	codon	
embA	Rv3794	545	codon	
embA	Rv3794	776	codon	
embA	Rv3794	777	codon	
embA	Rv3794	781	codon	
embA	Rv3794	782	codon	
embA	Rv3794	783	codon	
embA	Rv3794	784	codon	
embB	Rv3795	45	codon	
embB	Rv3795	228	codon	
embB	Rv3795	229	codon	
embB	Rv3795	230	codon	
embB	Rv3795	231	codon	
embB	Rv3795	232	codon	
embB	Rv3795	233	codon	
embB	Rv3795	234	codon	
embB	Rv3795	235	codon	
embB	Rv3795	236	codon	
embB	Rv3795	237	codon	
embB	Rv3795	238	codon	
embB	Rv3795	239	codon	ethambutol
embB	Rv3795	240	codon	ethambutol
embB	Rv3795	241	codon	
embB	Rv3795	242	codon	
embB	Rv3795	243	codon	
embB	Rv3795	244	codon	
embB	Rv3795	245	codon	
embB	Rv3795	246	codon	
embB	Rv3795	247	codon	

embB	Rv3795	248	codon
embB	Rv3795	249	codon
embB	Rv3795	250	codon
embB	Rv3795	251	codon
embB	Rv3795	252	codon
embB	Rv3795	749	codon
embB	Rv3795	785	codon
embB	Rv3795	786	codon
embB	Rv3795	787	codon
embB	Rv3795	788	codon
embB	Rv3795	789	codon
embB	Rv3795	918	codon
embB	Rv3795	919	codon
embB	Rv3795	920	codon
embB	Rv3795	921	codon

Analysis pipeline specifications

Pipeline version	3.0.4
Database version	tbdb_b2af444_Jody Phelan <jody.phelan@lshtm.ac.uk>_Mon Dec 21 06:39:13 2020 +0000
Analysis	Program
Mapping	bwa
Variant Calling	freebayes

Drugs.Drug
ciprofloxacin,fluoroquinolones,levofloxacin,moxifloxacin,ofloxacin
ciprofloxacin,fluoroquinolones,levofloxacin,moxifloxacin,ofloxacin
rifampicin
streptomycin
streptomycin
amikacin,aminoglycosides,capreomycin,kanamycin
amikacin,aminoglycosides,capreomycin,kanamycin
isoniazid
pyrazinamide
ethambutol

on
