# Supplementary material for: Whole genome sequencing of clinical samples reveals extensively drug resistant tuberculosis (XDR TB) strains from the Beijing lineage in Nigeria, West Africa
Source: Sci Rep. 2021 Aug 30;11:17387. doi: 10.1038/s41598-021-96956-7 (PMC8405707; doi:10.1038/s41598-021-96956-7)
Supplement: Supplementary file 4 — Supplementary Information 4. [file 41598_2021_96956_MOESM4_ESM.pdf]

{
species": {
"susceptibility": {
"Ofloxacin": {
"predict": "R

called_by": {
"gyrA_A90V-GCG7569GTG": {
"variant": null,
"genotype": [
1,
1
],
"genotype_likelihoods": [
-4156.2683044349105,
-99999999,
-33.62396202487085
],
"info": {
"coverage": {
"reference": {
"percent_coverage": 0.0,
"median_depth": 0,
"min_non_zero_depth": 0,
"kmer_count": 0,
"klen": 21
},
"alternate": {
"percent_coverage": 100.0,
"median_depth": 35,
"min_non_zero_depth": 29,
"kmer_count": 645,
"klen": 20
}
},
"expected_depths": [
36.0
],
"contamination_depths": [],
"filter": [],
"conf": 4123
},
"_cls": "Call.VariantCall

}

gyrA_S91P-TCG7572CCG": {
"variant": null,
"genotype": [
1,
1
],
"genotype_likelihoods": [
-4149.614044931797,
-99999999,
-33.30358561967774
],
"info": {
"coverage": {
"reference": {
"percent_coverage": 0.0,
"median_depth": 0,
"min_non_zero_depth": 0,
"kmer_count": 0,
"klen": 21
},
"alternate": {
"percent_coverage": 100.0,
"median_depth": 34,
"min_non_zero_depth": 26,
"kmer_count": 651,
"klen": 21
}
},
"expected_depths": [
36.0
],
"contamination_depths": [],
"filter": [],
"conf": 4116
},
"_cls": "Call.VariantCall
}
}
}
Moxifloxacin": {
"predict": "R

called_by": {
"gyrA_A90V-GCG7569GTG": {
"variant": null,
"genotype": [
1,
1
],
"genotype_likelihoods": [
-4156.2683044349105,
-99999999,
-33.62396202487085
],
"info": {
"coverage": {
"reference": {
"percent_coverage": 0.0,
"median_depth": 0,
"min_non_zero_depth": 0,
"kmer_count": 0,
"klen": 21
},
"alternate": {
"percent_coverage": 100.0,
"median_depth": 35,
"min_non_zero_depth": 29,
"kmer_count": 645,
"klen": 20
}
},
"expected_depths": [
36.0
],
"contamination_depths": [],
"filter": [],
"conf": 4123
},
"_cls": "Call.VariantCall
}

gyrA_S91P-TCG7572CCG": {
"variant": null,
"genotype": [
1,
1
],
"genotype_likelihoods": [
-4149.614044931797,
-99999999,
-33.30358561967774
],
"info": {
"coverage": {
"reference": {
"percent_coverage": 0.0,
"median_depth": 0,
"min_non_zero_depth": 0,
"kmer_count": 0,
"klen": 21
},
"alternate": {
"percent_coverage": 100.0,
"median_depth": 34,
"min_non_zero_depth": 26,
"kmer_count": 651,
"klen": 21
}
},
"expected_depths": [
36.0
],
"contamination_depths": [],
"filter": [],
"conf": 4116
},
"_cls": "Call.VariantCall
}
}
}
Isoniazid": {
"predict": "R

called_by": {
"katG_S315G-GCT2155167GGT": {
"variant": null,
"genotype": [
1,
1
],
"genotype_likelihoods": [
-4152.2847937542065,
-99999999,
-33.78276631168106
],
"info": {
"coverage": {
"reference": {
"percent_coverage": 0.0,
"median_depth": 0,
"min_non_zero_depth": 0,
"kmer_count": 0,
"klen": 21
},
"alternate": {
"percent_coverage": 100.0,
"median_depth": 33,
"min_non_zero_depth": 30,
"kmer_count": 644,
"klen": 20
}
},
"expected_depths": [
36.0
],
"contamination_depths": [],
"filter": [],
"conf": 4119
},
"_cls": "Call.VariantCall
}
}
}
Kanamycin": {
"predict": "S

}
Ethambutol": {
"predict": "R

called_by": {
"embB_Q497R-CAG4248002CGG": {
"variant": null,
"genotype": [
1,
1
],
"genotype_likelihoods": [
-5530.242242144776,
-99999999,
-53.822196462405216
],
"info": {
"coverage": {
"reference": {
"percent_coverage": 10.0,
"median_depth": 0,
"min_non_zero_depth": 3,
"kmer_count": 6,
"klen": 21
},
"alternate": {
"percent_coverage": 100.0,
"median_depth": 54,
"min_non_zero_depth": 46,
"kmer_count": 1012,
"klen": 20
}
},
"expected_depths": [
36.0
],
"contamination_depths": [],
"filter": [],
"conf": 5476
},
"_cls": "Call.VariantCall
}
}

}
Streptomycin": {
"predict": "R

called_by": {
"rpsL_K43R-AAG781686AGG": {
"variant": null,
"genotype": [
1,
1
],
"genotype_likelihoods": [
-3332.827451025354,
-99999999,
-60.49961361237543
],
"info": {
"coverage": {
"reference": {
"percent_coverage": 15.0,
"median_depth": 0,
"min_non_zero_depth": 8,
"kmer_count": 25,
"klen": 21
},
"alternate": {
"percent_coverage": 100.0,
"median_depth": 26,
"min_non_zero_depth": 19,
"kmer_count": 516,
"klen": 20
}
},
"expected_depths": [
36.0
],
"contamination_depths": [],
"filter": [],
"conf": 3272
},
"_cls": "Call.VariantCall
}
}

}
Ciprofloxacin": {
"predict": "R

called_by": {
"gyrA_A90V-GCG7569GTG": {
"variant": null,
"genotype": [
1,
1
],
"genotype_likelihoods": [
-4156.2683044349105,
-99999999,
-33.62396202487085
],
"info": {
"coverage": {
"reference": {
"percent_coverage": 0.0,
"median_depth": 0,
"min_non_zero_depth": 0,
"kmer_count": 0,
"klen": 21
},
"alternate": {
"percent_coverage": 100.0,
"median_depth": 35,
"min_non_zero_depth": 29,
"kmer_count": 645,
"klen": 20
}
},
"expected_depths": [
36.0
],
"contamination_depths": [],
"filter": [],
"conf": 4123
},
"_cls": "Call.VariantCall
}

gyrA_S91P-TCG7572CCG": {
"variant": null,
"genotype": [
1,
1
],
"genotype_likelihoods": [
-4149.614044931797,
-99999999,
-33.30358561967774
],
"info": {
"coverage": {
"reference": {
"percent_coverage": 0.0,
"median_depth": 0,
"min_non_zero_depth": 0,
"kmer_count": 0,
"klen": 21
},
"alternate": {
"percent_coverage": 100.0,
"median_depth": 34,
"min_non_zero_depth": 26,
"kmer_count": 651,
"klen": 21
}
},
"expected_depths": [
36.0
],
"contamination_depths": [],
"filter": [],
"conf": 4116
},
"_cls": "Call.VariantCall
}
}
}
Pyrazinamide": {
"predict": "R

called_by": {
"pncA_D12G-GTC2289206GCC": {
"variant": null,
"genotype": [
1,
1
],
"genotype_likelihoods": [
-2652.19858517686,
-401.66263875697615,
-38.94665231200829
],
"info": {
"coverage": {
"reference": {
"percent_coverage": 100.0,
"median_depth": 1,
"min_non_zero_depth": 1,
"kmer_count": 20,
"klen": 21
},
"alternate": {
"percent_coverage": 100.0,
"median_depth": 34,
"min_non_zero_depth": 30,
"kmer_count": 651,
"klen": 20
}
},
"expected_depths": [
36.0
],
"contamination_depths": [],
"filter": [],
"conf": 2613
},
"_cls": "Call.VariantCall
}
}
}
Rifampicin": {
"predict": "R

called_by": {
"rpoB_S450L-TCG761154TTG": {
"variant": null,
"genotype": [
1,
1
],
"genotype_likelihoods": [
-3943.441351392887,
-99999999,
-44.48201722857098
],
"info": {
"coverage": {
"reference": {
"percent_coverage": 0.0,
"median_depth": 0,
"min_non_zero_depth": 0,
"kmer_count": 0,
"klen": 21
},
"alternate": {
"percent_coverage": 100.0,
"median_depth": 31,
"min_non_zero_depth": 27,
"kmer_count": 591,
"klen": 20
}
},
"expected_depths": [
36.0
],
"contamination_depths": [],
"filter": [],
"conf": 3899
},
"_cls": "Call.VariantCall
}
}
}
Amikacin": {
"predict": "S

}
Capreomycin": {
"predict": "S
}
}

phylogenetics": {
"phylo_group": {
"Mycobacterium_tuberculosis_complex": {
"percent_coverage": 99.535,
"median_depth": 36.0
}
},
"sub_complex": {
"Unknown": {
"percent_coverage": -1,
"median_depth": -1
}
},
"species": {
"Mycobacterium_tuberculosis": {
"percent_coverage": 98.417,
"median_depth": 33.0
}
},
"lineage": {
"lineage": [
"lineage2.2
]

```
calls_summary": {
          "lineage2.2": {
            "good_nodes": 2,
            "tree_depth": 2,
            "genotypes": {
              "lineage2": 1,
              "lineage2.2": 1
            }
          }
        },
        "calls": {
          "lineage2.2": {
            "lineage2": {
              "G497491A": {
                "variant": "ref-G497491A?var_name=G497491A&num_alts=1&ref=NC_000962.3&enum=0&gene=NA&mut=G497491A
```

```
genotype": [
            1,
            1
        ],
        "genotype_likelihoods": [
            -3281.7536496128914,
            -99999999,
            -115.1501435425712
        ],
        "info": {
            "coverage": {
                "reference": {
                    "percent_coverage": 0.0,
                    "median_depth": 0,
                    "min_non_zero_depth": 0,
                    "kmer_count": 0,
                    "klen": 21
                },
                "alternate": {
                    "percent_coverage": 100.0,
                    "median_depth": 20,
                    "min_non_zero_depth": 19,
                    "kmer_count": 419,
                    "klen": 21
                }
            },
            "expected_depths": [
                36.0
            ],
            "contamination_depths": [],
            "filter": [],
            "conf": 3167
        },
        "_cls": "Call.VariantCall
    }
}
lineage2.2": {
    "G2505085A": {
        "variant": "ref-G2505085A?var_name=G2505085A&num_alts=2&ref=NC_000962.3&enum=0&gene=NA&mut=G2505085A
```

```
genotype": [
                        1,
                        1
                    ],
                    "genotype_likelihoods": [
                        -3441.791097177168,
                        -99999999,
                        -90.97719069356732
                    ],
                    "info": {
                        "coverage": {
                            "reference": {
                                "percent_coverage": 0.0,
                                "median_depth": 0,
                                "min_non_zero_depth": 0,
                                "kmer_count": 0,
                                "klen": 21
                            },
                            "alternate": {
                                "percent_coverage": 100.0,
                                "median_depth": 23,
                                "min_non_zero_depth": 22,
                                "kmer_count": 464,
                                "klen": 21
                            }
                        },
                        "expected_depths": [
                            36.0
                        ],
                        "contamination_depths": [],
                        "filter": [],
                        "conf": 3351
                    },
                    "_cls": "Call.VariantCall
                }
            }
        }
    }
}
}
```

kmer": 21,
"probe_sets": [
"/home/idowu/miniconda3/lib/python3.6/site-packages/mykrobe/data/tb/tb-species-170421.fasta.gz
/home/idowu/miniconda3/lib/python3.6/site-packages/mykrobe/data/tb/tb-hunt-probe-set-jan-03-2019.fasta.gz
/home/idowu/miniconda3/lib/python3.6/site-packages/mykrobe/data/tb/tb.lineage.20200930.probes.fa.gz
]
files": [
"TB8qc_R1.fastq.gz
TB8qc_R2.fastq.gz
]
version": {
"mykrobe-predictor": "v0.9.0
mykrobe-atlas": "v0.9.0
}
genotype_model": "kmer_count
}
}
