# Supplementary material for: Whole genome sequencing of clinical samples reveals extensively drug resistant tuberculosis (XDR TB) strains from the Beijing lineage in Nigeria, West Africa
Source: Sci Rep. 2021 Aug 30;11:17387. doi: 10.1038/s41598-021-96956-7 (PMC8405707; doi:10.1038/s41598-021-96956-7)
Supplement: Supplementary file 5 — Supplementary Information 5. [file 41598_2021_96956_MOESM5_ESM.pdf]

TBProfiler report

Summary

ID	TB8_tbprofiler
Date	Mon May 3 15:58:24 2021
Strain	lineage2.2.1
Drug-resistance	XDR

Lineage report

Lineage	Estimated Fraction	Family	Spoligotype	Rd
lineage2	0.993	East-Asian	Beijing	RD105
lineage2.2	0.985	East-Asian (Beijing)	Beijing-RD207	RD105;RD207
lineage2.2.1	0.987	East-Asian (Beijing)	Beijing-RD181	RD105;RD207;RD181

Resistance report

Drug	Genotypic Resistance	Mutations
Rifampicin	R	rpoB p.Ser450Leu (1.00)
Isoniazid	R	katG c.1124 1125insC (0.13), katG p.Ser315Thr (1.00)
Ethambutol	R	embB p.Gln497Arg (1.00)
Pyrazinamide	R	pncA p.Asp12Gly (0.97)
Streptomycin	R	rpsL p.Lys43Arg (1.00), rrs r.799c>t (0.45), rrs r.888g>a (0.31)
Fluoroquinolones	R	gyrA p.Ala90Val (1.00), gyrA p.Ser91Pro (1.00)
Amikacin	R	rrs r.1402c>a (0.27), rrs r.1484g>t (0.13)
Capreomycin	R	rrs r.1402c>a (0.27), rrs r.1484g>t (0.13)
Kanamycin	R	rrs r.1402c>a (0.27), rrs r.1484g>t (0.13)
Cycloserine		
Ethionamide		
Clofazimine		
Para-aminosalicylic acid		

Delamanid
Bedaquiline
Linezolid

Resistance variants report

Genome Position	Locus Tag	Gene	Change	Estimated Fraction	Drugs.Drug
7570	Rv0006	gyrA	p.Ala90Val	1	ciprofloxacin,fluoroquinolo
7572	Rv0006	gyrA	p.Ser91Pro	1	ciprofloxacin,fluoroquinolo
761155	Rv0667	rpoB	p.Ser450Leu	1	rifampicin
781687	Rv0682	rpsL	p.Lys43Arg	1	streptomycin
1472644	rrs	rrs	r.799c>t	0.451	streptomycin
1472733	rrs	rrs	r.888g>a	0.308	streptomycin
1473247	rrs	rrs	r.1402c>a	0.275	amikacin,aminoglycosides
1473329	rrs	rrs	r.1484g>t	0.127	amikacin,aminoglycosides
2154987	Rv1908c	katG	c.1124 1125insC	0.133	isoniazid
2155168	Rv1908c	katG	p.Ser315Thr	1	isoniazid
2289207	Rv2043c	pncA	p.Asp12Gly	0.973	pyrazinamide
4248003	Rv3795	embB	p.Gln497Arg	1	ethambutol

Other variants report

Genome Position	Locus Tag	Change	Estimated Fraction
5509	Rv0005	c.270C>A	0.133
6316	Rv0005	c.1077G>C	0.087
6571	Rv0005	c.1332T>C	0.091
7362	Rv0006	p.Glu21Gln	1
7585	Rv0006	p.Ser95Thr	1
7965	Rv0006	c.664C>A	0.087
8576	Rv0006	c.1275C>A	0.167
8840	Rv0006	c.1539C>G	0.111
8844	Rv0006	p.Asp515Asn	0.105
8849	Rv0006	c.1548C>G	0.111

8852	Rv0006	c.1551T>C	0.1
8858	Rv0006	c.1557T>G	0.087
8912	Rv0006	p.Gln537His	0.091
8920	Rv0006	p.Gly540Asp	0.087
8941	Rv0006	c.1641 1641del	0.1
9304	Rv0006	p.Gly668Asp	1
491080	Rv0407	p.Arg100Cys	0.091
491107	Rv0407	p.Glu109*	0.095
491180	Rv0407	p.Arg133Leu	0.091
491237	Rv0407	p.Gly152Val	0.095
491383	Rv0407	p.Glu201*	0.087
491742	Rv0407	c.960T>C	1
575566	Rv0486	c.82G>T	0.1
575588	Rv0486	p.Arg81Cys	0.105
575633	Rv0486	p.Ala96Thr	0.091
575907	Rv0486	p.Ala187Val	1
576442	Rv0486	p.Cys365*	0.1
576580	Rv0486	c.1097 1097del	0.087
576663	Rv0486	p.Ala439Asp	0.095
759372	Rv0667	c.-435G>C	0.1
759553	Rv0667	c.-254G>A	0.133
759983	Rv0667	p.Ile59Met	0.1
760916	Rv0667	c.1110C>T	0.136
760928	Rv0667	c.1122G>C	0.087
760944	Rv0667	p.Gln380Glu	0.087
760958	Rv0667	c.1152G>C	0.174
760959	Rv0667	p.Val385Thr	0.13
760965	Rv0667	p.Met387Leu	0.2
760970	Rv0667	c.1164G>C	0.2
760982	Rv0667	c.1176G>C	0.136
760985	Rv0667	c.1179G>C	0.136
760988	Rv0667	c.1182C>G	0.105
760991	Rv0667	c.1185G>C	0.176
760997	Rv0667	c.1191G>C	0.118
761015	Rv0667	c.1209G>C	0.267

761027	Rv0667	c.1221A>C	0.2
761036	Rv0667	c.1230G>C	0.2
761037	Rv0667	c.1231T>C	0.2
761233	Rv0667	p.Arg476Gln	0.087
761564	Rv0667	c.1758G>T	0.105
762003	Rv0667	p.Asn733Gln	0.103
762009	Rv0667	p.Leu735Met	0.111
762015	Rv0667	p.Glu737Ser	0.111
762029	Rv0667	c.2223C>G	0.13
762101	Rv0667	c.2295C>G	0.1
762114	Rv0667	p.Ile770Val	0.095
762117	Rv0667	p.Ser771Gly	0.1
762125	Rv0667	p.Glu773Asp	0.091
762131	Rv0667	c.2325C>G	0.095
762137	Rv0667	c.2331C>T	0.095
762143	Rv0667	c.2337T>C	0.095
762148	Rv0667	p.Arg781Leu	0.091
762151	Rv0667	p.Gly782Asp	0.105
762156	Rv0667	p.Val784Ile	0.1
762167	Rv0667	c.2361T>C	0.105
762174	Rv0667	c.2369 2369del	0.118
762179	Rv0667	c.2373C>A	0.118
762181	Rv0667	p.Asp792Ala	0.118
762194	Rv0667	c.2388G>C	0.095
762206	Rv0667	c.2400C>G	0.111
762209	Rv0667	c.2403C>G	0.222
762218	Rv0667	c.2412T>C	0.25
762233	Rv0667	c.2427G>C	0.154
762236	Rv0667	c.2430G>C	0.154
762251	Rv0667	c.2445G>C	0.167
762254	Rv0667	c.2448T>C	0.167
762257	Rv0667	c.2451C>G	0.143
762266	Rv0667	c.2460T>C	0.2
762857	Rv0667	c.3051C>G	0.143
762863	Rv0667	c.3057T>G	0.118

762878	Rv0667	p.Ile1024Met	0.133
762879	Rv0667	p.Met1025Leu	0.167
763031	Rv0667	c.3225T>C	0.958
763070	Rv0667	c.3264T>C	0.105
763075	Rv0667	p.Thr1090Ile	0.1
763468	Rv0668	c.99G>C	0.087
763483	Rv0668	c.114G>C	0.208
763486	Rv0668	c.117T>G	0.208
763507	Rv0668	c.138G>C	0.13
763528	Rv0668	c.159G>C	0.174
763531	Rv0668	c.162G>C	0.115
763534	Rv0668	c.165T>C	0.231
763537	Rv0668	c.168C>G	0.208
763546	Rv0668	c.177A>G	0.267
763550	Rv0668	p.Tyr61Ala	0.129
763570	Rv0668	c.201G>C	0.217
763573	Rv0668	c.204G>C	0.167
763594	Rv0668	c.225C>T	0.107
763622	Rv0668	p.Ala85Ser	0.261
763627	Rv0668	p.Lys86Asn	0.174
763633	Rv0668	c.264T>C	0.208
763636	Rv0668	c.267T>C	0.182
763642	Rv0668	c.273G>C	0.238
763660	Rv0668	c.291T>C	0.111
763669	Rv0668	c.300C>G	0.217
763696	Rv0668	c.327T>C	0.143
763708	Rv0668	c.339G>C	0.143
763714	Rv0668	c.345G>C	0.118
763717	Rv0668	c.348T>C	0.133
763720	Rv0668	c.351G>A	0.133
763732	Rv0668	c.363C>G	0.143
763744	Rv0668	c.375G>C	0.111
763751	Rv0668	p.Ile128Val	0.1
763765	Rv0668	c.396T>G	0.091
764018	Rv0668	p.Asp217Tyr	0.133

764308	Rv0668	c.939G>C	0.182
764317	Rv0668	c.948C>T	0.111
764318	Rv0668	p.Val317Ile	0.111
764338	Rv0668	p.Glu323Asp	0.1
764344	Rv0668	c.975C>T	0.136
764365	Rv0668	c.996C>T	0.138
764371	Rv0668	c.1002G>C	0.1
764380	Rv0668	c.1011G>C	0.143
764387	Rv0668	c.1018T>C	0.162
764431	Rv0668	c.1062G>C	0.088
764434	Rv0668	c.1065A>G	0.091
764441	Rv0668	p.Ile358Leu	0.091
764461	Rv0668	c.1092A>G	0.111
764497	Rv0668	c.1128A>G	0.186
764498	Rv0668	p.Ser377Ala	0.195
764503	Rv0668	c.1134G>C	0.195
764507	Rv0668	p.Ala380Ser	0.2
764521	Rv0668	c.1152T>C	0.359
764527	Rv0668	c.1158C>T	0.135
764536	Rv0668	c.1167G>T	0.192
764539	Rv0668	c.1170C>G	0.3
764545	Rv0668	c.1176C>G	0.107
764548	Rv0668	c.1179G>C	0.292
764560	Rv0668	c.1191T>C	0.182
764566	Rv0668	c.1197C>G	0.194
764572	Rv0668	c.1203G>C	0.111
764575	Rv0668	c.1206T>G	0.226
764578	Rv0668	c.1209C>G	0.118
764581	Rv0668	c.1212T>C	0.389
764582	Rv0668	p.Leu405Met	0.297
764605	Rv0668	c.1236G>C	0.184
764611	Rv0668	c.1242G>T	0.226
764623	Rv0668	c.1254C>G	0.184
764626	Rv0668	c.1257C>T	0.105
764632	Rv0668	c.1263T>C	0.238

764635	Rv0668	c.1266C>G	0.156
764644	Rv0668	c.1275G>C	0.24
764650	Rv0668	c.1281G>T	0.19
764665	Rv0668	c.1296C>G	0.093
764695	Rv0668	c.1326T>C	0.12
764706	Rv0668	p.Leu446Gln	0.213
764713	Rv0668	c.1344G>C	0.104
764746	Rv0668	c.1377G>C	0.103
764749	Rv0668	c.1380G>C	0.156
764758	Rv0668	c.1389C>G	0.089
764764	Rv0668	c.1395T>C	0.162
764780	Rv0668	c.1411A>T	0.139
764824	Rv0668	c.1455T>C	0.107
764849	Rv0668	p.His494Asn	0.091
764858	Rv0668	c.1489T>C	0.15
764916	Rv0668	p.Leu516Pro	0.913
765051	Rv0668	p.Ser561Cys	0.087
766218	Rv0668	p.Asp950Gly	0.133
766661	Rv0668	p.Val1098Leu	0.25
766690	Rv0668	c.3321G>T	0.154
779154	Rv0678	c.165A>G	0.105
779164	Rv0678	p.Ala59Thr	0.095
779221	Rv0678	p.Gly78Arg	0.105
779270	Rv0678	p.Arg94Leu	0.111
779298	Rv0678	c.309C>T	0.118
779302	Rv0678	p.Arg105Ser	0.133
781395	Rv0682	c.-165T>C	1
781594	Rv0682	p.Arg12Leu	0.1
781655	Rv0682	c.96T>C	0.087
781775	Rv0682	p.His72Gln	0.087
800802	Rv0701	c.-7C>A	0.088
800839	Rv0701	p.Leu11Met	0.118
1303844	Rv1173	p.Ala305Asp	0.13
1303962	Rv1173	c.1032G>T	0.125
1304229	Rv1173	p.Met433Ile	0.091

1304271	Rv1173	c.1341C>A	0.111
1304807	Rv1173	p.Pro626Arg	0.095
1304908	Rv1173	p.Gly660Trp	0.143
1416521	Rv1267c	p.Val276Gly	0.095
1416968	Rv1267c	p.Ala127Asp	0.143
1417202	Rv1267c	p.Pro49Leu	0.087
1461038	Rv1305	c.-7T>C	0.118
1471922	rrs	r.77gt>g	0.096
1471925	rrs	r.80t>c	0.094
1472106	rrs	r.261g>a	0.263
1472108	rrs	r.263c>t	0.27
1472112	rrs	r.267c>t	0.105
1472123	rrs	r.278a>t	0.211
1472150	rrs	r.305t>a	0.346
1472151	rrs	r.306c>a	0.326
1472160	rrs	r.315c>t	0.203
1472164	rrs	r.319g>a	0.121
1472172	rrs	r.327t>c	0.492
1472177	rrs	r.332c>t	0.175
1472203	rrs	r.358g>a	0.196
1472210	rrs	r.365a>c	0.167
1472213	rrs	r.368g>c	0.125
1472214	rrs	r.369c>g	0.123
1472215	rrs	r.370a>g	0.151
1472225	rrs	r.380c>a	0.152
1472234	rrs	r.389t>c	0.118
1472235	rrs	r.390g>c	0.098
1472240	rrs	r.395g>a	0.154
1472251	rrs	r.406g>a	0.3
1472259	rrs	r.414c>a	0.143
1472278	rrs	r.433c>t	0.1
1472279	rrs	r.434t>c	0.094
1472286	rrs	r.441c>g	0.129
1472289	rrs	r.444t>g	0.241
1472290	rrs	r.445c>g	0.233

1472293	rrs	r.448c>a	0.1
1472324	rrs	r.479g>c	0.25
1472325	rrs	r.480g>c	0.25
1472328	rrs	r.483g>c	0.174
1472338	rrs	r.493a>g	0.111
1472344	rrs	r.499c>t	0.455
1472382	rrs	r.537g>a	0.212
1472389	rrs	r.544g>a	0.194
1472400	rrs	r.555c>t	0.188
1472435	rrs	r.590t>c	0.152
1472438	rrs	r.593t>c	0.091
1472439	rrs	r.594c>t	0.152
1472448	rrs	r.603t>c	0.143
1472450	rrs	r.605a>g	0.143
1472452	rrs	r.607g>a	0.108
1472462	rrs	r.617t>c	0.143
1472464	rrs	r.619a>g	0.212
1472471	rrs	r.626g>a	0.161
1472489	rrs	r.644a>t	0.154
1472494	rrs	r.649a>g	0.128
1472498	rrs	r.653c>t	0.357
1472530	rrs	r.685g>a	0.292
1472557	rrs	r.712g>a	0.139
1472558	rrs	r.713g>a	0.444
1472569	rrs	r.724g>a	0.414
1472570	rrs	r.725g>a	0.114
1472571	rrs	r.726g>c	0.121
1472573	rrs	r.728c>t	0.171
1472579	rrs	r.734g>t	0.11
1472581	rrs	r.736a>t	0.25
1472584	rrs	r.739a>t	0.238
1472596	rrs	r.751g>t	0.145
1472598	rrs	r.753a>t	0.15
1472607	rrs	r.762g>a	0.43
1472612	rrs	r.767g>t	0.258

1472614	rrs	r.769g>t	0.151
1472616	rrs	r.771g>a	0.181
1472655	rrs	r.810g>t	0.2
1472658	rrs	r.813g>a	0.095
1472660	rrs	r.815t>c	0.474
1472661	rrs	r.816a>g	0.434
1472692	rrs	r.847t>c	0.107
1472695	rrs	r.850c>t	0.13
1472697	rrs	r.852t>c	0.167
1472713	rrs	r.868t>c	0.586
1472714	rrs	r.869a>g	0.463
1472716	rrs	r.871c>t	0.257
1472742	rrs	r.897c>t	0.344
1472744	rrs	r.899a>g	0.379
1472767	rrs	r.922g>a	0.371
1472779	rrs	r.934g>a	0.123
1472781	rrs	r.936c>t	0.438
1472790	rrs	r.945t>c	0.115
1472793	rrs	r.948a>t	0.407
1472803	rrs	r.958t>a	0.211
1472824	rrs	r.979t>a	0.1
1472825	rrs	r.980g>a	0.1
1472828	rrs	r.983t>c	0.395
1472836	rrs	r.991g>a	0.129
1472844	rrs	r.999c>t	0.091
1472845	rrs	r.1000g>c	0.097
1472846	rrs	r.1001c>g	0.097
1472848	rrs	r.1003t>g	0.103
1472859	rrs	r.1014g>t	0.097
1472861	rrs	r.1016g>t	0.103
1472874	rrs	r.1029c>t	0.175
1472875	rrs	r.1030t>g	0.146
1472880	rrs	r.1035g>a	0.295
1472895	rrs	r.1050c>t	0.451
1472952	rrs	r.1107t>c	0.221

1472955	rrs	r.1110c>t	0.258
1472956	rrs	r.1111t>c	0.369
1472957	rrs	r.1112c>t	0.508
1472973	rrs	r.1128a>g	0.231
1472974	rrs	r.1129a>t	0.14
1472977	rrs	r.1132g>gt	0.138
1472987	rrs	r.1142g>a	0.526
1472988	rrs	r.1143t>a	0.111
1472989	rrs	r.1144g>a	0.164
1472990	rrs	r.1145a>g	0.323
1473002	rrs	r.1157g>t	0.111
1473004	rrs	r.1159t>a	0.125
1473005	rrs	r.1160c>t	0.173
1473009	rrs	r.1164t>c	0.148
1473026	rrs	r.1181t>c	0.15
1473035	rrs	r.1190g>a	0.389
1473051	rrs	r.1206t>c	0.107
1473055	rrs	r.1210c>t	0.147
1473056	rrs	r.1211a>t	0.235
1473062	rrs	r.1217t>a	0.108
1473066	rrs	r.1221a>g	0.188
1473080	rrs	r.1235c>t	0.323
1473088	rrs	r.1243a>g	0.189
1473089	rrs	r.1244a>c	0.156
1473091	rrs	r.1246g>c	0.122
1473093	rrs	r.1248c>t	0.19
1473099	rrs	r.1254t>c	0.128
1473100	rrs	r.1255g>a	0.325
1473102	rrs	r.1257c>t	0.238
1473104	rrs	r.1259c>t	0.357
1473110	rrs	r.1265t>g	0.326
1473111	rrs	r.1266a>g	0.37
1473115	rrs	r.1270g>t	0.158
1473121	rrs	r.1276t>c	0.286
1473122	rrs	r.1277t>a	0.114

1473123	rrs	r.1278a>t	0.188
1473132	rrs	r.1287t>c	0.152
1473135	rrs	r.1290c>t	0.189
1473145	rrs	r.1300c>t	0.556
1473147	rrs	r.1302g>t	0.122
1473148	rrs	r.1303g>a	0.186
1473163	rrs	r.1318c>t	0.267
1473164	rrs	r.1319c>a	0.149
1473166	rrs	r.1321g>a	0.636
1473173	rrs	r.1328c>t	0.122
1473177	rrs	r.1332g>a	0.211
1473191	rrs	r.1346c>t	0.14
1473206	rrs	r.1361g>a	0.178
1473226	rrs	r.1381c>t	0.182
1473248	rrs	r.1403g>a	0.102
1473249	rrs	r.1404t>c	0.102
1473252	rrs	r.1407t>c	0.412
1473255	rrs	r.1410a>g	0.098
1473259	rrs	r.1414c>t	0.451
1473260	rrs	r.1415g>t	0.098
1473276	rrs	r.1431a>g	0.304
1473283	rrs	r.1438t>c	0.257
1473301	rrs	r.1456t>c	0.233
1473315	rrs	r.1470t>c	0.122
1473316	rrs	r.1471c>t	0.104
1473352	rrs	r.1507c>t	0.13
1473427	rrl	c.-231G>T	0.095
1474112	rrl	r.455t>g	0.087
1474124	rrl	r.467g>c	0.233
1474125	rrl	r.468c>g	0.233
1474130	rrl	r.473c>t	0.125
1474135	rrl	r.478g>a	0.167
1474140	rrl	r.483c>t	0.351
1474141	rrl	r.484g>c	0.189
1474142	rrl	r.485c>g	0.2

1474151	rrl	r.494c>t	0.417
1474155	rrl	r.498g>a	0.25
1474164	rrl	r.507c>t	0.297
1474174	rrl	r.517a>g	0.209
1474181	rrl	r.524c>t	0.265
1474183	rrl	r.526t>c	0.344
1474184	rrl	r.527c>t	0.382
1474197	rrl	r.540c>t	0.146
1474202	rrl	r.545t>c	0.256
1474249	rrl	r.592g>t	0.37
1474253	rrl	r.596a>t	0.244
1474263	rrl	r.606g>a	0.324
1474266	rrl	r.609t>c	0.088
1474269	rrl	r.612c>t	0.294
1474311	rrl	r.654g>gt	0.107
1474348	rrl	r.691c>t	0.194
1474351	rrl	r.694g>c	0.292
1474353	rrl	r.696a>g	0.304
1474354	rrl	r.697c>t	0.138
1474362	rrl	r.705a>g	0.267
1474365	rrl	r.708g>a	0.111
1474384	rrl	r.727c>t	0.182
1474387	rrl	r.730c>t	0.294
1474402	rrl	r.745t>c	0.138
1474467	rrl	r.810a>g	0.152
1474483	rrl	r.826c>g	0.091
1474488	rrl	r.831g>t	0.2
1474496	rrl	r.839c>t	0.105
1474497	rrl	r.840g>c	0.22
1474498	rrl	r.841g>t	0.146
1474505	rrl	r.848c>g	0.146
1474506	rrl	r.849c>g	0.209
1474507	rrl	r.850g>t	0.116
1474516	rrl	r.859c>a	0.314
1474527	rrl	r.870t>c	0.127

1474529	rrl	r.872a>c	0.191
1474530	rrl	r.873g>a	0.125
1474537	rrl	r.880g>a	0.482
1474539	rrl	r.882c>t	0.125
1474540	rrl	r.883t>g	0.146
1474551	rrl	r.894g>c	0.135
1474552	rrl	r.895c>t	0.115
1474558	rrl	r.901g>a	0.146
1474626	rrl	r.969t>c	0.25
1474627	rrl	r.970g>a	0.107
1474632	rrl	r.975g>t	0.344
1474634	rrl	r.977t>g	0.107
1474636	rrl	r.979a>t	0.321
1474637	rrl	r.980c>t	0.321
1474639	rrl	r.982g>c	0.333
1474663	rrl	r.1006c>t	0.143
1474676	rrl	r.1019t>a	0.219
1474692	rrl	r.1035g>a	0.238
1474709	rrl	r.1052gtggt>g	0.125
1474734	rrl	r.1077g>t	0.286
1474736	rrl	r.1079c>t	0.179
1474749	rrl	r.1092c>t	0.323
1474751	rrl	r.1094g>a	0.259
1474753	rrl	r.1096ac>a	0.435
1474760	rrl	r.1103a>g	0.423
1474777	rrl	r.1120t>c	0.167
1474779	rrl	r.1122g>a	0.227
1474780	rrl	r.1123c>t	0.091
1474782	rrl	r.1125g>a	0.136
1474783	rrl	r.1126g>a	0.174
1474784	rrl	r.1127c>t	0.32
1474794	rrl	r.1137c>t	0.654
1474798	rrl	r.1141c>g	0.323
1474803	rrl	r.1146g>a	0.176
1474812	rrl	r.1155g>a	0.326

1474823	rrl	r.1166c>g	0.5
1474824	rrl	r.1167a>g	0.212
1474825	rrl	r.1168g>a	0.288
1474827	rrl	r.1170c>t	0.192
1474830	rrl	r.1173a>g	0.326
1474831	rrl	r.1174a>c	0.111
1474832	rrl	r.1175a>t	0.105
1474837	rrl	r.1180a>g	0.269
1474839	rrl	r.1182c>t	0.141
1474844	rrl	r.1187g>t	0.167
1474864	rrl	r.1207c>t	0.433
1474866	rrl	r.1209c>a	0.247
1474869	rrl	r.1212g>t	0.356
1474892	rrl	r.1235g>a	0.353
1474896	rrl	r.1239a>g	0.6
1474901	rrl	r.1244a>g	0.255
1474902	rrl	r.1245t>c	0.34
1474903	rrl	r.1246t>a	0.311
1474904	rrl	r.1247g>c	0.659
1474905	rrl	r.1248t>c	0.326
1474913	rrl	r.1256t>c	0.436
1474917	rrl	r.1260g>t	0.128
1474918	rrl	r.1261t>c	0.128
1474920	rrl	r.1263g>c	0.378
1474921	rrl	r.1264c>t	0.297
1474932	rrl	r.1275c>t	0.4
1474938	rrl	r.1281g>a	0.25
1475057	rrl	r.1400g>a	0.125
1475060	rrl	r.1403ac>a	0.158
1475063	rrl	r.1406a>t	0.15
1475076	rrl	r.1419c>t	0.143
1475088	rrl	r.1431a>g	0.13
1475090	rrl	r.1433a>t	0.13
1475094	rrl	r.1437c>t	0.13
1475108	rrl	r.1451c>t	0.2

1475110	rrl	r.1453aggcc>a	0.231
1475118	rrl	r.1461c>t	0.176
1475120	rrl	r.1463g>gtctc	0.2
1475129	rrl	r.1472g>t	0.211
1475137	rrl	r.1480a>t	0.15
1475170	rrl	r.1513a>t	0.238
1475173	rrl	r.1516a>t	0.095
1475190	rrl	r.1533t>a	0.182
1475368	rrl	r.1711c>a	0.087
1475380	rrl	r.1723c>a	0.1
1475419	rrl	r.1762c>t	0.105
1475499	rrl	r.1842c>t	0.1
1475514	rrl	r.1857g>a	0.111
1475526	rrl	r.1869c>a	0.217
1475531	rrl	r.1874c>t	0.208
1475539	rrl	r.1882a>t	0.16
1475544	rrl	r.1887a>t	0.111
1475545	rrl	r.1888t>g	0.148
1475550	rrl	r.1893a>c	0.133
1475552	rrl	r.1895g>c	0.133
1475672	rrl	r.2015c>t	0.1
1475673	rrl	r.2016t>c	0.107
1475696	rrl	r.2039t>a	0.118
1475697	rrl	r.2040c>t	0.088
1475699	rrl	r.2042c>t	0.269
1475703	rrl	r.2046a>t	0.118
1475704	rrl	r.2047c>t	0.143
1475713	rrl	r.2056c>g	0.154
1475716	rrl	r.2059a>g	0.138
1475722	rrl	r.2065g>t	0.294
1475747	rrl	r.2090a>g	0.1
1475753	rrl	r.2096c>t	0.222
1475766	rrl	r.2109g>c	0.188
1475769	rrl	r.2112t>c	0.308
1475775	rrl	r.2118g>t	0.143

1475781 rrl	r.2124t>c	0.109
1475803 rrl	r.2146t>c	0.169
1475804 rrl	r.2147g>c	0.167
1475816 rrl	r.2159c>g	0.205
1475817 rrl	r.2160a>g	0.229
1475858 rrl	r.2201t>c	0.124
1475869 rrl	r.2212c>a	0.157
1475879 rrl	r.2222t>c	0.096
1475881 rrl	r.2224t>c	0.346
1475883 rrl	r.2226a>t	0.413
1475884 rrl	r.2227a>g	0.321
1475897 rrl	r.2240t>c	0.378
1475898 rrl	r.2241a>g	0.271
1475899 rrl	r.2242g>a	0.235
1475900 rrl	r.2243a>g	0.202
1475902 rrl	r.2245t>c	0.229
1475906 rrl	r.2249c>t	0.271
1475916 rrl	r.2259c>g	0.216
1475937 rrl	r.2280a>t	0.136
1475943 rrl	r.2286g>a	0.267
1475952 rrl	r.2295a>g	0.306
1475970 rrl	r.2313c>t	0.177
1475975 rrl	r.2318c>t	0.378
1475977 rrl	r.2320a>g	0.394
1475978 rrl	r.2321c>t	0.106
1475988 rrl	r.2331a>g	0.383
1475993 rrl	r.2336c>t	0.186
1475997 rrl	r.2340a>t	0.151
1476001 rrl	r.2344t>c	0.097
1476131 rrl	r.2474c>t	0.154
1476160 rrl	r.2503t>c	0.133
1476164 rrl	r.2507a>g	0.102
1476194 rrl	r.2537a>g	0.306
1476200 rrl	r.2543a>t	0.28
1476201 rrl	r.2544c>t	0.25

1476214 rrl	r.2557g>t	0.104
1476215 rrl	r.2558c>t	0.167
1476224 rrl	r.2567a>g	0.212
1476225 rrl	r.2568t>g	0.182
1476229 rrl	r.2572c>t	0.221
1476245 rrl	r.2588c>t	0.186
1476251 rrl	r.2594t>c	0.383
1476252 rrl	r.2595t>g	0.167
1476256 rrl	r.2599a>t	0.157
1476260 rrl	r.2603a>g	0.582
1476268 rrl	r.2611a>t	0.304
1476275 rrl	r.2618t>a	0.302
1476279 rrl	r.2622g>a	0.281
1476280 rrl	r.2623a>c	0.316
1476281 rrl	r.2624t>c	0.246
1476293 rrl	r.2636c>t	0.327
1476294 rrl	r.2637a>g	0.327
1476295 rrl	r.2638c>g	0.362
1476296 rrl	r.2639c>t	0.353
1476297 rrl	r.2640c>t	0.457
1476298 rrl	r.2641c>a	0.109
1476300 rrl	r.2643g>t	0.163
1476301 rrl	r.2644a>c	0.357
1476302 rrl	r.2645g>a	0.254
1476309 rrl	r.2652g>c	0.107
1476311 rrl	r.2654g>c	0.383
1476312 rrl	r.2655t>c	0.379
1476313 rrl	r.2656g>a	0.377
1476332 rrl	r.2675g>c	0.372
1476353 rrl	r.2696g>t	0.451
1476358 rrl	r.2701t>c	0.511
1476359 rrl	r.2702c>g	0.182
1476369 rrl	r.2712c>t	0.523
1476372 rrl	r.2715t>c	0.487
1476381 rrl	r.2724g>c	0.185

1476382	rrl	r.2725a>g	0.5
1476383	rrl	r.2726t>a	0.493
1476408	rrl	r.2751g>a	0.526
1476425	rrl	r.2768g>t	0.455
1476428	rrl	r.2771c>t	0.796
1476429	rrl	r.2772a>c	0.531
1476466	rrl	r.2809c>t	0.589
1476481	rrl	r.2824t>c	0.724
1476506	rrl	r.2849t>c	0.598
1476514	rrl	r.2857c>t	0.233
1476515	rrl	r.2858c>t	0.193
1476517	rrl	r.2860c>t	0.245
1476519	rrl	r.2862c>g	0.16
1476530	rrl	r.2873c>t	0.259
1476535	rrl	r.2878g>a	0.192
1476536	rrl	r.2879g>a	0.431
1476537	rrl	r.2880a>g	0.176
1476538	rrl	r.2881a>g	0.294
1476539	rrl	r.2882a>g	0.392
1476540	rrl	r.2883c>g	0.52
1476547	rrl	r.2890c>t	0.29
1476567	rrl	r.2910c>t	0.38
1476572	rrl	r.2915g>a	0.265
1476573	rrl	r.2916a>t	0.438
1476584	rrl	r.2927c>t	0.424
1476585	rrl	r.2928a>g	0.091
1476592	rrl	r.2935g>a	0.154
1476605	rrl	r.2948c>t	0.095
1476607	rrl	r.2950c>t	0.095
1476622	rrl	r.2965g>t	0.091
1673380	Rv1483	c.-60C>G	0.1
1674892	Rv1484	p.Asn231Asp	1
1833414	Rv1630	c.-128AT>A	0.095
1833727	Rv1630	c.186G>C	0.125
1833742	Rv1630	c.201A>G	0.118

1833745	Rv1630	c.204G>C	0.111
1833797	Rv1630	p.Val86Ile	0.087
1833811	Rv1630	c.270G>C	0.091
1833829	Rv1630	c.288A>G	0.125
1833832	Rv1630	c.291G>A	0.125
1833838	Rv1630	c.297G>C	0.107
1833841	Rv1630	c.300C>G	0.154
1833847	Rv1630	c.306C>G	0.148
1833856	Rv1630	c.315A>G	0.174
1833862	Rv1630	c.321G>T	0.125
1833894	Rv1630	p.Ala118Glu	0.12
1833928	Rv1630	c.387G>C	0.111
1833949	Rv1630	c.408T>C	0.12
1833970	Rv1630	c.429G>C	0.103
1833979	Rv1630	c.438T>C	0.115
1833988	Rv1630	c.447C>G	0.111
1833997	Rv1630	c.456G>C	0.094
1834015	Rv1630	c.474G>C	0.088
1834069	Rv1630	c.528G>C	0.111
1834177	Rv1630	c.636A>C	1
1834249	Rv1630	c.708T>C	0.125
1834261	Rv1630	c.720A>G	0.111
1834264	Rv1630	c.723G>C	0.103
1834486	Rv1630	p.Glu315Asp	0.091
1834489	Rv1630	c.948T>C	0.143
1834528	Rv1630	c.987T>C	0.143
1917972	Rv1694	c.33A>G	1
1918255	Rv1694	p.Ala106Ser	0.091
2154898	Rv1908c	p.Ala405Asp	0.105
2155051	Rv1908c	p.Thr354Ile	0.154
2155218	Rv1908c	p.Leu298Phe	0.1
2155389	Rv1908c	c.723G>T	0.182
2155930	Rv1908c	p.Ala61Glu	0.1
2518234	Rv2245	p.Glu40Asp	0.111
2518273	Rv2245	p.Lys53Asn	0.105

2518470	Rv2245	p.Ala119Asp	0.095
2518535	Rv2245	p.Ala141Thr	0.1
2518554	Rv2245	p.Pro147Gln	0.087
2518809	Rv2245	p.Lys232Arg	0.089
2519094	Rv2245	p.Arg327Leu	0.1
2714131	Rv2416c	p.Glu401Gly	0.182
2714136	Rv2416c	c.1197C>A	0.182
2714967	Rv2416c	p.Glu122Asp	0.087
2715005	Rv2416c	p.Asp110Tyr	0.154
2746242	Rv2447c	p.Asp453Tyr	0.118
2746641	Rv2447c	p.Gln320Lys	0.111
2746681	Rv2447c	c.918G>T	0.1
2746705	Rv2447c	p.Gln298His	0.118
2746710	Rv2447c	p.His297Asn	0.105
2746789	Rv2447c	p.Ile270Met	0.133
2746900	Rv2447c	c.699G>T	0.091
2859422	Rv2535c	p.Arg333Cys	0.087
2859808	Rv2535c	p.His204Pro	0.118
2859877	Rv2535c	p.Leu181Pro	0.182
2987357	Rv2671	c.519C>A	0.111
2987369	Rv2671	c.531G>T	0.111
2987462	Rv2671	c.624C>A	0.105
2987526	Rv2671	p.Gly230Leu	0.1
2987533	Rv2671	p.Gly232Val	0.095
3067517	Rv2754c	p.Tyr143*	0.111
3067540	Rv2754c	p.Ala136Ser	0.125
3067924	Rv2754c	p.Arg8Cys	0.118
3067985	Rv2754c	c.-40C>A	0.111
3068083	Rv2754c	c.-138T>C	0.118
3068091	Rv2754c	c.-146T>G	0.118
3086788	Rv2780	c.-32T>C	1
3087318	Rv2780	p.Glu167*	0.133
3087726	Rv2780	p.Ala303Ser	0.1
3087767	Rv2780	c.948G>T	0.1
3087809	Rv2780	c.990G>T	0.118

3339519	Rv2983	p.His134Gln	0.095
3339608	Rv2983	p.Pro164Gln	0.111
3339646	Rv2983	p.Glu177*	0.118
3339650	Rv2983	p.Leu178Pro	0.125
3640722	Rv3261	c.180G>T	0.105
3640817	Rv3261	p.Leu92Pro	0.087
3641077	Rv3261	p.Gln179Lys	0.105
3641082	Rv3261	c.540G>T	0.1
3641206	Rv3261	p.Ala222Thr	0.091
3641598	Rv3262	p.Glu22*	0.087
3641666	Rv3262	p.Asp44Glu	0.1
3641715	Rv3262	p.Val61Phe	0.105
3641794	Rv3262	p.Ala87Glu	0.125
3642035	Rv3262	c.501T>C	0.1
3642197	Rv3262	c.663C>T	0.111
3642308	Rv3262	c.774G>T	0.111
3642339	Rv3262	p.Asp269Tyr	0.111
3841187	Rv3423c	c.234G>T	0.095
3841662	Rv3423c	c.-242A>G	0.198
3841663	Rv3423c	c.-243G>A	0.092
3841677	Rv3423c	c.-257C>T	0.176
3841679	Rv3423c	c.-259T>G	0.165
3841694	Rv3423c	c.-274C>A	0.152
4044212	Rv3601c	p.Gly24Cys	0.1
4044248	Rv3601c	p.Arg12Ser	0.105
4240866	Rv3793	p.Ala335Asp	0.1
4240982	Rv3793	p.Ser374Gly	0.111
4241347	Rv3793	c.1485C>A	0.087
4241526	Rv3793	p.Gly555Val	0.105
4241678	Rv3793	p.Val606Ile	0.182
4242096	Rv3793	p.Ala745Asp	0.2
4242643	Rv3793	c.2781C>T	1
4242651	Rv3793	p.Arg930Leu	0.125
4242735	Rv3793	p.Ile958Thr	0.118
4243115	Rv3793	c.3253C>T	0.133

4243460	Rv3794	c.228C>T	1
4244339	Rv3794	c.1107G>T	0.105
4244574	Rv3794	p.Ala448Ser	0.118
4244642	Rv3794	c.1410G>T	0.125
4244845	Rv3794	c.1613 1614insC	0.167
4244981	Rv3794	c.1749G>T	0.091
4245581	Rv3794	c.2349C>A	0.125
4245600	Rv3794	p.Pro790Thr	0.111
4245962	Rv3794	p.Gln910His	0.091
4246238	Rv3794	c.3006T>C	0.105
4246544	Rv3795	p.Thr11Pro	0.37
4246548	Rv3795	p.Pro12Gln	0.2
4246555	Rv3795	c.42G>C	0.25
4246556	Rv3795	p.Ala15Pro	0.25
4246567	Rv3795	c.54 55insT	0.286
4246712	Rv3795	p.Val67Leu	0.143
4246897	Rv3795	c.384C>A	0.188
4247018	Rv3795	p.Gly169Cys	0.118
4247269	Rv3795	c.756C>A	0.095
4247739	Rv3795	p.Ala409Glu	0.143
4247938	Rv3795	c.1425G>T	0.091
4248616	Rv3795	c.2103G>C	0.095
4248882	Rv3795	p.Pro790His	0.095
4248942	Rv3795	p.Gly810Val	0.118
4249141	Rv3795	c.2628G>T	0.091
4249162	Rv3795	c.2649C>A	0.095
4249331	Rv3795	p.Pro940Thr	0.105
4249362	Rv3795	p.Ala950Asp	0.111
4249757	Rv3795	p.Thr1082Ala	1
4269022	Rv3806c	p.Ala271Asp	0.105
4269116	Rv3806c	p.Arg240Cys	1
4326064	Rv3854c	c.1410G>T	0.111
4326125	Rv3854c	p.Gly450Val	0.182
4326405	Rv3854c	p.Asp357Tyr	1
4326744	Rv3854c	c.730G>A	0.091

4326967	Rv3854c	c.507C>T	0.133
4327375	Rv3854c	c.99G>C	0.095
4327443	Rv3854c	p.Gly11Cys	0.091
4327591	Rv3855	p.Arg15Trp	0.091
4327989	Rv3855	c.441C>A	0.105
4328055	Rv3855	p.His169Gln	0.125
4407588	Rv3919c	c.615T>C	1
4407605	Rv3919c	p.Ala200Thr	0.133
4407634	Rv3919c	p.Thr190Lys	0.091
4407813	Rv3919c	c.390G>T	0.111
4407850	Rv3919c	p.Arg118His	0.118
4407927	Rv3919c	p.Glu92Asp	1
4408243	Rv3919c	c.-41G>T	0.1

Coverage report

Gene	Locus_Tag	Cutoff	Fraction
gyrB	Rv0005	0	0
gyrA	Rv0006	0	0
fgd1	Rv0407	0	0
mshA	Rv0486	0	0
rpoB	Rv0667	0	0
rpoC	Rv0668	0	0
mmpR5	Rv0678	0	0
rpsL	Rv0682	0	0
rplC	Rv0701	0	0
fbiC	Rv1173	0	0
embR	Rv1267c	0	0
atpE	Rv1305	0	0
rrs	rrs	0	0
rrl	rrl	0	0
fabG1	Rv1483	0	0
inhA	Rv1484	0	0
rpsA	Rv1630	0	0

tlyA	Rv1694	0	0
katG	Rv1908c	0	0
pncA	Rv2043c	0	0
kasA	Rv2245	0	0
eis	Rv2416c	0	0
ahpC	Rv2428	0	0
folC	Rv2447c	0	0
pepQ	Rv2535c	0	0
ribD	Rv2671	0	0
thyX	Rv2754c	0	0
thyA	Rv2764c	0	0
ald	Rv2780	0	0
fbiD	Rv2983	0	0
fbiA	Rv3261	0	0
fbiB	Rv3262	0	0
alr	Rv3423c	0	0
ddn	Rv3547	0	0
panD	Rv3601c	0	0
embC	Rv3793	0	0
embA	Rv3794	0	0
embB	Rv3795	0	0
ubiA	Rv3806c	0	0
ethA	Rv3854c	0	0
ethR	Rv3855	0	0
gid	Rv3919c	0	0

Missing positions report

Gene	Locus_Tag	Position	Position_Type	Drug_Resistance_Position
rpoB	Rv0667	202	codon	
rpoB	Rv0667	203	codon	
rpoB	Rv0667	204	codon	
rpoB	Rv0667	205	codon	
rpoB	Rv0667	206	codon	

rpoB	Rv0667	209	codon
rpoB	Rv0667	211	codon
rpoB	Rv0667	212	codon
rpoB	Rv0667	213	codon
rpoB	Rv0667	214	codon
rpoB	Rv0667	215	codon
rpoC	Rv0668	288	codon
rpoC	Rv0668	289	codon
rpoC	Rv0668	290	codon
atpE	Rv1305	18	codon
atpE	Rv1305	19	codon
atpE	Rv1305	20	codon
atpE	Rv1305	21	codon
atpE	Rv1305	22	codon
atpE	Rv1305	23	codon
atpE	Rv1305	24	codon
rrl	rrl	1295	gene
rrl	rrl	1296	gene
rrl	rrl	1297	gene
rrl	rrl	1298	gene
rrl	rrl	1299	gene
rrl	rrl	1300	gene
rrl	rrl	1301	gene
rrl	rrl	1302	gene
rrl	rrl	1303	gene
rrl	rrl	1304	gene
rrl	rrl	1305	gene
rrl	rrl	1306	gene
rrl	rrl	1307	gene
rrl	rrl	1308	gene
rrl	rrl	1309	gene
rrl	rrl	1310	gene
rrl	rrl	1311	gene
rrl	rrl	1312	gene
rrl	rrl	1313	gene

rrl	rrl	1314	gene
rrl	rrl	1315	gene
rrl	rrl	1316	gene
rrl	rrl	1317	gene
rrl	rrl	1318	gene
rrl	rrl	1319	gene
rrl	rrl	1320	gene
rrl	rrl	1321	gene
rrl	rrl	1322	gene
rrl	rrl	1323	gene
rrl	rrl	1324	gene
rrl	rrl	1325	gene
rrl	rrl	1326	gene
rrl	rrl	1327	gene
rrl	rrl	1328	gene
rrl	rrl	1329	gene
rrl	rrl	1330	gene
rrl	rrl	1331	gene
rrl	rrl	1332	gene
rrl	rrl	1333	gene
rrl	rrl	1334	gene
rrl	rrl	1335	gene
rrl	rrl	1336	gene
rrl	rrl	1337	gene
rrl	rrl	1338	gene
rrl	rrl	1339	gene
rrl	rrl	1340	gene
rrl	rrl	1341	gene
rrl	rrl	1342	gene
rrl	rrl	1343	gene
rrl	rrl	1344	gene
rrl	rrl	1345	gene
rrl	rrl	1346	gene
rrl	rrl	1347	gene
rrl	rrl	1348	gene

rrl	rrl	1349	gene	
rrl	rrl	1350	gene	
rrl	rrl	1351	gene	
rrl	rrl	1352	gene	
rrl	rrl	1353	gene	
rrl	rrl	1354	gene	
rrl	rrl	1355	gene	
rrl	rrl	1356	gene	
rrl	rrl	1357	gene	
rrl	rrl	1358	gene	
rrl	rrl	1359	gene	
rrl	rrl	1624	gene	
rrl	rrl	1635	gene	
rrl	rrl	1636	gene	
rrl	rrl	1637	gene	
rrl	rrl	1638	gene	
katG	Rv1908c	265	codon	isoniazid
katG	Rv1908c	264	codon	isoniazid
katG	Rv1908c	263	codon	
katG	Rv1908c	262	codon	isoniazid
katG	Rv1908c	261	codon	isoniazid
katG	Rv1908c	260	codon	
katG	Rv1908c	259	codon	isoniazid
katG	Rv1908c	258	codon	isoniazid
katG	Rv1908c	257	codon	isoniazid
katG	Rv1908c	256	codon	
katG	Rv1908c	255	codon	
katG	Rv1908c	254	codon	
katG	Rv1908c	253	codon	
katG	Rv1908c	252	codon	isoniazid
eis	Rv2416c	101	codon	
eis	Rv2416c	100	codon	
eis	Rv2416c	99	codon	
pepQ	Rv2535c	178	codon	
pepQ	Rv2535c	177	codon	

pepQ	Rv2535c	176 codon
pepQ	Rv2535c	175 codon
pepQ	Rv2535c	174 codon
embC	Rv3793	421 codon
embC	Rv3793	422 codon
embC	Rv3793	423 codon
embC	Rv3793	424 codon
embC	Rv3793	425 codon
embC	Rv3793	426 codon
embC	Rv3793	427 codon
embC	Rv3793	428 codon
embC	Rv3793	429 codon
embC	Rv3793	430 codon
embC	Rv3793	431 codon
embC	Rv3793	432 codon
embC	Rv3793	433 codon
embC	Rv3793	434 codon
embC	Rv3793	436 codon
embC	Rv3793	437 codon
embA	Rv3794	547 codon
embA	Rv3794	548 codon
embA	Rv3794	552 codon
embA	Rv3794	553 codon
embA	Rv3794	554 codon
embA	Rv3794	555 codon

Analysis pipeline specifications

Pipeline version	3.0.4
Database version	tbdb_b2af444_Jody Phelan <jody.phelan@lshtm.ac.uk>_Mon Dec 21 06:39:13 2020 +0000
Analysis	Program
Mapping	bwa
Variant Calling	freebayes

nes,levofloxacin,moxifloxacin,ofloxacin
nes,levofloxacin,moxifloxacin,ofloxacin

,capreomycin,kanamycin
,capreomycin,kanamycin
